# Emulating a target trial of surgical removal of uterine fibroids on atherosclerotic cardiovascular disease

**DOI:** 10.1016/j.fertnstert.2026.05.151

**Published:** 2026-05-19

**Authors:** Julia D. DiTosto, Jennifer Lewey, Jarcy Zee, Anuja Dokras, Kyle R. Busse, Snigdha Alur-Gupta, Stefanie N. Hinkle, Enrique F. Schisterman, Sunni L. Mumford, Ellen C. Caniglia

**Affiliations:** a Department of Biostatistics, Epidemiology, and Informatics, Perelman School of Medicine, University of Pennsylvania, Philadelphia, Pennsylvania;; b Division of Cardiovascular Medicine, University of Pennsylvania Perelman School of Medicine, Philadelphia, Pennsylvania;; c Children’s Hospital of Philadelphia Research Institute, Philadelphia, Pennsylvania;; d Department of Obstetrics and Gynecology, University of Pennsylvania Perelman School of Medicine, Philadelphia, Pennsylvania;; e Department of Obstetrics and Gynecology, University of Rochester Medical Center, Rochester, New York

**Keywords:** Uterine fibroids, target trial emulation, cardiovascular disease, hysterectomy, myomectomy

## Abstract

**Objective:**

To emulate a target trial comparing surgical removal (hysterectomy, myomectomy, or no surgery) of uterine fibroids on atherosclerotic cardiovascular disease (CVD) risk.

**Design:**

Retrospective cohort study leveraging Optum’s deidentified Clinformatics Data Mart Database in the United States (2000–2024).

**Subjects:**

Eligible individuals were females aged 18–50 with codes for uterine fibroids. Individuals were followed until an atherosclerotic CVD event, disenrollment in the healthcare plan, or 11/1/2024.

**Exposure:**

Sequential monthly “trials” classified individuals as undergoing myomectomy, hysterectomy (without oophorectomy, which has distinct indications), or no surgery during 2000–2019.

**Main Outcome Measures:**

The 5-year risk of atherosclerotic CVD was estimated as a composite of coronary artery disease, cerebrovascular disease, and peripheral artery disease. Individual components and events (e.g., myocardial infarction) were evaluated. Baseline confounding by age, cardiovascular risk factors, reproductive and mental health was adjusted for using inverse probability weighting (IPW). Exploratory and subgroup analyses by surgical route, considering oophorectomy, race and ethnicity, and age were conducted. Additional models accounted for subsequent surgeries using time-varying IPW.

**Results:**

Analysis included 11,106 individuals with uterine fibroids who underwent myomectomy, 23,089 hysterectomy, and 27,657 no surgery. Compared with no surgery, myomectomy was not associated with 5-year atherosclerotic CVD risk (adjusted risk difference [aRD] −0.93 percentage points, 95% confidence interval [CI]: −2.04–1.30) whereas hysterectomy was associated with a 1.24 percentage-point higher risk (95% CI: −0.01–2.03). Analyses accounting for subsequent surgeries were consistent. Hysterectomy was associated with increased risk of coronary artery disease (0.88 percentage points, 95% CI: 0.03–1.66) and myocardial infarction (0.52 percentage points, 95% CI: 0.02–1.05). In exploratory analyses, open/abdominal hysterectomy was associated with a 2.75 percentage point higher atherosclerotic CVD risk (95% CI: 0.94–4.06). Among individuals aged <40 years, hysterectomy was associated with a 1.86 percentage point increased 5-year risk, although imprecise (95% CI: −0.35–3.08).

**Conclusions:**

Myomectomy was not strongly associated with atherosclerotic CVD risk in individuals with uterine fibroids, whereas hysterectomy (without oophorectomy) was modestly associated with a higher 5-year atherosclerotic CVD risk, particularly among younger individuals. These associations warrant further investigation.

## Abstract (es)

**Objetivo:**

Emular un ensayo objetivo para comparar el efecto de la resección quirúrgica de los miomas uterinos (histerectomía, miomectomía o ausencia de cirugía) sobre el riesgo de enfermedad cardiovascular (ECV) aterosclerótica.

**Diseño:**

Estudio de cohorte retrospectivo utilizando la base de datos desidentificada Optum Clinformatics Data Mart de Estados Unidos (2000–2024).

**Sujetos:**

Mujeres de 18 a 50 años con códigos diagnósticos de miomas uterinos. Las participantes fueron seguidas hasta la ocurrencia de un evento de ECV aterosclerótica, la pérdida de afiliación al plan de salud o el 1 de noviembre de 2024.

**Exposición:**

Se emularon “ensayos” mensuales secuenciales, clasificando a las participantes según se sometieran a miomectomía, histerectomía (sin ooforectomía, la cual tiene indicaciones distintas) o ausencia de cirugía durante el periodo 2000–2019.

**Medidas principales de resultado:**

Se estimó el riesgo de ECV aterosclerótica a 5 años como un desenlace compuesto de enfermedad arterial coronaria, enfermedad cerebrovascular y enfermedad arterial periférica. También se evaluaron los componentes individuales y eventos específicos (p. ej., infarto de miocardio). Se ajustó por factores de confusión basales relacionados con la edad, factores de riesgo cardiovascular y antecedentes reproductivos y de salud mental mediante ponderación por el inverso de la probabilidad (IPW). Se realizaron análisis exploratorios y de subgrupos según la vía quirúrgica, considerando la ooforectomía, la raza y la etnia, y la edad. Modelos adicionales incorporaron las cirugías subsecuentes mediante IPW dependiente del tiempo.

**Resultados:**

El análisis incluyó a 11,106 mujeres con miomas uterinos sometidas a miomectomía, 23,089 a histerectomía y 27,657 sin tratamiento quirúrgico. En comparación con el grupo sin cirugía, la miomectomía no se asoció con el riesgo de ECV aterosclerótica a 5 años (diferencia de riesgo ajustada [aRD] −0.93 puntos porcentuales; intervalo de confianza [IC] del 95%: −2.04 a 1.30), mientras que la histerectomía se asoció con un incremento de 1.24 puntos porcentuales en el riesgo (IC del 95%: −0.01 a 2.03). Los análisis que consideraron las cirugías subsecuentes mostraron resultados consistentes. La histerectomía se asoció con un mayor riesgo de enfermedad arterial coronaria (0.88 puntos porcentuales; IC del 95%: 0.03 a 1.66) e infarto de miocardio (0.52 puntos porcentuales; IC del 95%: 0.02 a 1.05). En los análisis exploratorios, la histerectomía abdominal se asoció con un incremento de 2.75 puntos porcentuales en el riesgo de ECV aterosclerótica (IC del 95%: 0.94 a 4.06). Entre las mujeres menores de 40 años, la histerectomía se asoció con un incremento de 1.86 puntos porcentuales en el riesgo a 5 años, aunque la estimación fue imprecisa (IC del 95%: −0.35 a 3.08).

**Conclusiones:**

La miomectomía no se asoció de forma importante con el riesgo de ECV aterosclerótica en mujeres con miomas uterinos, mientras que la histerectomía (sin ooforectomía) se asoció modestamente con un mayor riesgo de ECV aterosclerótica a 5 años, particularmente en mujeres más jóvenes. Estos hallazgos justifican investigaciones adicionales.

Uerine fibroids (leiomyomas) are common benign gynecologic tumors affecting over 26 million reproductive-aged individuals in the United States (1, 2). Atherosclerotic cardiovascular disease (CVD), encompassing coronary artery disease, cerebrovascular disease, and peripheral artery disease (3), is the leading cause of death among women in the United States, accounting for approximately one in five female deaths annually (4). Recently, atherosclerotic CVD has been hypothesized to be linked to gynecologic conditions (5–7), including uterine fibroids (8). Uterine fibroids have been associated with a significantly higher atherosclerotic CVD risk, even after controlling for conventional risk factors (9–14). Proposed mechanisms include smooth muscle cell proliferation and hormonal alterations of uterine fibroids that may accelerate atherosclerosis through systemic inflammation and plaque formation (12, 13).

Nearly 25% of individuals with uterine fibroids undergo surgical removal through hysterectomy or myomectomy (15–19). Research examining the cardiovascular implications of surgical interventions for fibroids has primarily focused on hysterectomy (20–28), which is a procedure that removes the uterus. Studies among reproductive-aged individuals undergoing hysterectomy for indications nonspecific to fibroids have produced mixed results, with most finding associations with early-onset atherosclerotic CVD (20–25), hypothesized to be due to premature hormonal disruption or decreased ovarian blood flow (29). Others showed null associations (26–28). Myomectomy, which is a procedure that removes fibroids while preserving the uterus, is the second most common surgical intervention for fibroids (30). Unlike hysterectomy, myomectomy may preserve uterine blood flow and hormonal function (31, 32), potentially resulting in different cardiovascular effects, yet this has not been evaluated.

Understanding the cardiovascular effects of surgical interventions for fibroids is critical to inform treatment decisions for patients and their providers. Given the ethical and practical constraints of randomized controlled trials (RCTs) of surgical interventions, alternative approaches are needed. Target trial emulation is an analytic framework leveraging observational data to mimic a hypothetical RCT, reducing common biases in observational studies (33–35). The objective of this study was to leverage a claims database and target trial emulation to compare atherosclerotic CVD outcomes after surgical removal of uterine fibroids. We hypothesized that, compared with no surgery, undergoing a hysterectomy would be associated with an increased risk of atherosclerotic CVD, whereas undergoing a myomectomy would be associated with a decreased risk.

## MATERIALS AND METHODS

### Study data

We used target trial emulation to estimate the effect of surgical removal of fibroids on atherosclerotic CVD risk using observational data (36–39). When few individuals initiate the treatment at any one point in time, this approach can be extended to emulate a sequence of target trials to reduce selection bias and immortal time bias (39), producing estimates comparable with trials (33, 40).

The analysis utilized Optum’s deidentified Clinformatics Data Mart Database (Optum CDM) (2000–2024) (Supplemental Appendix 1, available online). Variables were identified using International Classification of Diseases (ICD)-9/10, Current Procedural Terminology (CPT), Healthcare Common Procedure Coding System (HCPCS), and National Drug Codes (NDC) (Supplemental Table 1, available online).

Informed consent was waived, given the use of fully deidentified data and minimal risk of harm. The study was reviewed and approved by the University of Pennsylvania Institutional Review Board.

### Eligibility criteria

The trial emulation protocol is described in Supplemental Table 2 (41).

Eligible individuals were females in Optum CDM, aged 18–50 years, with a diagnosis of uterine fibroids between December 1, 2000 and October 1, 2019 (allowing at least 5 years of follow-up), defined as ≥1 fibroid claim preceded by a transvaginal ultrasonography or pelvic magnetic resonance imaging claim within 30 days (Supplemental Table 1) (42, 43).

Individuals with a history of atherosclerotic CVD, hysterectomy, myomectomy, oophorectomy (unilateral or bilateral), menopause, or cancer were excluded. Those with hysterectomy and concurrent oophorectomy were excluded from the primary analysis, as the indications for oophorectomy are typically not related to uterine fibroids (44), but were considered in exploratory analyses. Pregnant or postpartum individuals were excluded, defined as 10 months before and 3 months after a delivery code.

To capture comorbidities before “trial” start, we required continuous health insurance coverage for ≥6 months before (30-day gap allowed) entry into the study cohort. We also required individuals to have a fibroid claim within 1 month of each trial start.

### Treatment arms and follow-up

To emulate a trial examining the effects of hysterectomy, myomectomy, or no surgery on incident atherosclerotic CVD, eligible individuals were followed until atherosclerotic CVD, disenrollment from Optum CDM, or November 2024. In the trial, death would be a censoring event, yet mortality data were unavailable.

Trials were defined on the calendar month (e.g., from December 1, 2000 to December 31, 2000). At baseline of the first trial, defined as December 2000, individuals were classified into the myomectomy arm if they had procedure codes for a myomectomy that month, the hysterectomy arm if they had procedure codes for a hysterectomy that month, and the no surgery arm otherwise. If both procedures were coded in the same month, they were coded in the hysterectomy group (2%) (30). To reduce computational time, we used a 5% random sample of the no surgery arm, resulting in a sample size comparable with other treatment groups.

We implemented a sequential target trial (36, 37, 39, 45–48), emulating 271 trials monthly from December 2000 through October 2019 (Supplemental Fig. 1, available online). Eligibility criteria were reassessed at each sequential trial. Individuals who were no longer eligible (i.e., because of age, comorbidity criteria or prior surgical removal) were excluded from that trial; all others were reclassified according to whether they underwent surgery during that month. Individuals could contribute to multiple trials to maximize statistical power (36, 37, 46).

### Causal contrasts

Trial emulation can yield observational analogues of the intention-to-treat (ITT) effect and the per-protocol effect. The observational analogue of the ITT effect was defined as the effect of undergoing (rather than being randomized) a treatment strategy at baseline. The observational analogue of the per-protocol effect was defined as the effect of undergoing a given treatment strategy and receiving no further intervention. The per-protocol effect is relevant because surgical reintervention after myomectomy is common (19, 30, 49).

### Outcomes

The primary outcome was 5-year risk of atherosclerotic CVD diagnosis or event, defined by the American Heart Association (3), including coronary artery disease (encompassing stable ischemic heart disease and acute myocardial infarction), cerebrovascular disease (including transient ischemic attack and ischemic stroke), and peripheral artery disease.

Secondary outcomes examined individual atherosclerotic CVD components and acute events. Outcomes were identified as the first ICD-9/10, CPT, or HCPCS claims. One inpatient or more than one outpatient claim was required for classification as an outcome (50, 51).

Cardiovascular risk factors, including incident type 2 diabetes, hyperlipidemia, hypertension, and obesity, were also examined, identified as the first ICD-9/10 or NDC code, if applicable. For these analyses, individuals with these conditions at baseline were excluded.

### Statistical analysis

Baseline variables were updated at the start of each trial (e.g., baseline variables for the December 2000 trial reflected the most recent measurements of covariates at that time).

To emulate randomization at baseline, inverse probability of treatment weights (IPTW) were estimated. Weights were fit across all trials with calendar time and trial number fit using natural splines. Variables used to generate weights included age at fibroid diagnosis and age at trial start (modeled using natural splines), and binary indicators of cardiovascular risk factors (obesity, hyperlipidemia, type 2 diabetes, hypertension, smoking), mental health (anxiety, depression), reproductive health (parity, infertility, endometriosis), fibroid symptoms (pelvic pain or heavy menstrual bleeding), and hormonal contraceptive use (oral and nonoral). Parity (parous vs. nulliparous) was defined as prior delivery in the dataset.

Use of tranexamic acid, gonadotropin-releasing hormone (GnRH) agonists, or prior procedural interventions was rare (<0.5%) but summarized descriptively. Balance after IPTW was assessed using standardized mean differences (SMD), with ≤0.1 considered acceptable (52).

For the ITT analysis, we fit pooled logistic regression models for each outcome and used predicted values to estimate 5-year risks, weighted by IPTW. Because the monthly event probability was small, the pooled logistic model parameters closely approximated a Cox proportional hazards model (53). Data from all trials were pooled into one model, including baseline month, follow-up month, and treatment-time interaction, modeled using natural splines.

The per-protocol analysis was similar, except individuals were censored at deviation from the assigned treatment strategy. Specifically, those in the myomectomy group were censored if and when they had a hysterectomy, and the no surgery group was censored if and when they had a hysterectomy or myomectomy. Because a hysterectomy removes the uterus, these individuals were never censored, even if coded with a myomectomy (likely coding error). To adjust for time-varying selection bias induced by artificial censoring, individuals received time-varying weights of the inverse probability of adhering to treatment. At each time point, individuals were weighted by the cumulative product of their IPTW and inverse probability of adhering to treatment. Additional details are provided in the Supplemental Appendix 2.

Weights were truncated at the 99th percentile. We calculated 95% confidence intervals (CIs) using bootstrapping (500 repetitions).

Missing data for the exposure, outcome, and confounders were not present, as binary indicators were defined as the presence or absence of a code. Analyses were performed in RStudio (Version 4.0.2, RStudio Team).

### Subgroup analyses

Race and ethnicity could not be included in the primary adjustment because data were unavailable for the full cohort, although they may be important covariates as indicators of structural factors (2, 54, 55). Among a subcohort (55.8%) with self-reported race and ethnicity data, stratified analyses (White, Black, Asian, Hispanic) were conducted. Given age differences in hormone production (56, 57), analyses were stratified by age at trial start (<40 years and ≥40 years). Because of small event counts, only ITT models were fit for stratified analyses. For each of these analyses, IPTW were refit in the stratified cohorts.

### Exploratory analyses

Two exploratory analyses were conducted. We examined the surgical route, given that minimally invasive approaches may reduce surgical stress and inflammation compared with open procedures (58–60). Route was classified as open/abdominal or minimally invasive (endoscopic, laparoscopic, vaginal) on the basis of procedure codes. Codes that did not specify route were excluded (myomectomy: 9.2%, N = 1,716; hysterectomy: 0.05%, N = 23).

Given the known association between oophorectomy and atherosclerotic CVD (20, 61–64), we included individuals who underwent a hysterectomy with unilateral oophorectomy in exploratory analyses. Although excluded from the primary analysis because clinical indications may differ from the indications for hysterectomy alone, some individuals may elect concurrent unilateral oophorectomy for other indications (e.g., risk of ovarian cancer) (44). Individuals with bilateral oophorectomy or codes for removing the “remaining” uterus remained excluded.

### Sensitivity analyses

We conducted several sensitivity analyses. First, we excluded individuals who used infertility medications at any time during follow-up as a proxy for baseline pregnancy intentions; such individuals would not enroll in a trial where hysterectomy was an option (58), although including them in the primary analysis would induce selection bias. Second, we computed inverse probability of censoring weights (IPCW) to adjust for selection bias because of loss to follow-up (Supplemental Appendix 2). For each month, the cumulative product of the IPTW and IPCW was used. Third, we extended the required baseline insurance coverage from ≥6 months to ≥12 months to see if capturing longer baseline data impacted results. Fourth, we note that hysteroscopic myomectomy (CPT 58561) was included within the minimally invasive category in the primary route analysis. Hysteroscopic myomectomy is clinically distinct from laparoscopic or robotic approaches and is typically performed for submucosal fibroids, which differ in number, size, and burden from fibroids treated via other minimally invasive routes. We conducted a sensitivity analysis separating hysteroscopic myomectomy from other minimally invasive approaches to examine whether this grouping influenced results.

## RESULTS

### Primary results

Among 1,076,918 individuals with uterine fibroids, 337,090 individuals were eligible across 589,848 person-months (Supplemental Fig. 2). The study included 11,106 myomectomy, 23,089 hysterectomy, and 27,657 no surgery individuals (5% random sample). Baseline characteristics are shown in Table 1; after weighting, all SMD were <0.1 (Supplemental Fig. 3).

Over an average follow-up of 2.41 years (myomectomy; SD = 1.73), 2.38 years (hysterectomy; SD1.73), and 2.44 years (no surgery; SD = 1.75), 149 atherosclerotic CVD diagnoses and events following myomectomy, 506 following hysterectomy, and 606 in the no surgery group occurred. Figure 1A shows the cumulative risk curves for risk of atherosclerotic CVD by treatment group.

At 5 years, individuals who underwent a myomectomy had a 0.93 percentage-point lower 5-year risk of atherosclerotic CVD (95% CI: −2.04, 1.30) compared with no surgery, although this was imprecise. Hysterectomy was associated with a 1.24 percentage-point increased 5-year risk of atherosclerotic CVD compared with no surgery (95% CI: −0.01, 2.03) (Table 2).

After 5-years of follow-up, 2,030 (7.34%) in the no surgery group underwent a myomectomy and 4,152 (15.01%) underwent a hysterectomy, whereas 563 (5.07%) in the myomectomy group underwent a hysterectomy. Results were similar for per-protocol analyses accounting for subsequent surgeries (Fig. 1B). Compared with no surgery, undergoing a myomectomy was associated with a 0.98 percentage-point reduction in atherosclerotic CVD (95% −2.10, 1.27) and hysterectomy a 1.39 percentage-point increase in atherosclerotic CVD (95% CI 0.02, 2.16) (Table 2).

### Secondary outcomes

Estimates for secondary outcomes of individual atherosclerotic CVD diagnoses and events were imprecise because of few events. There were no differences in the 5-year risk of coronary artery disease, myocardial infarction, transient ischemic attack, and peripheral artery disease after myomectomy compared with no surgery. Undergoing a myomectomy was associated with modestly decreased, although imprecise, risk of cerebrovascular disease (adjusted risk difference [aRD] −0.86%, 95% CI: −1.61, 0.66) and ischemic stroke (aRD −0.42%, 95% CI: −1.00, 0.55). Undergoing a hysterectomy was associated with modestly increased risk of coronary artery disease (aRD 0.88%, 95% CI: 0.03, 1.66), myocardial infarction (aRD 0.52%, 95% CI: 0.02, 1.05), and ischemic stroke (aRD 0.62%, 95% CI: −0.06, 1.23) (Fig. 2; Supplemental Table 3).

There were no differences in the 5-year risk of cardiovascular risk factors among those who underwent myomectomy compared with those who had no surgery. At 5 years, individuals who underwent hysterectomy had a higher risk of obesity (aRD 9.36%, 95% CI: 8.40, 11.73), hypertension (aRD 3.85%, 95% CI: 1.29, 5.62), and hyperlipidemia (aRD 4.45%, 95% CI: 1.49, 5.80) (Supplemental Table 3).

### Subgroup analyses

Among those with race and ethnicity data, 22,437 (65.0%) were White, 10,131 (29.3%) Black, 1,966 (5.7%) Asian, and 5,163 (14.9%) Hispanic. Estimates were imprecise across groups because of few events (Supplemental Table 4). Among those aged <40, hysterectomy was associated with an increased 5-year atherosclerotic CVD risk (aRD 1.86%, 95% CI: −0.35, 3.08), whereas myomectomy was associated with a decreased risk in the age ≥40 group (aRD −1.50%, 95% CI: −2.94, 0.74) (Supplemental Table 5).

### Exploratory analyses

Among myomectomies, 34.9% (N = 6,513) were open/abdominal and 55.9% (N = 10,451) were minimally invasive; for hysterectomy, 32.4% were open/abdominal (N = 13,740) and 67.5% (28,583) were minimally invasive. At 5 years, compared with no surgery, minimally invasive myomectomy was associated with a 1.69 percentage-point (95% CI: −2.91, 0.28) lower risk of atherosclerotic CVD. Open hysterectomy was associated with a 2.75 percentage-point higher risk (95% CI: 0.94, 4.06) (Supplemental Table 6).

Among 2,441 (9.6%) individuals who underwent hysterectomy with unilateral oophorectomy, 5-year atherosclerotic CVD risk was 2.06 percentage points higher (95% CI: −0.87, 4.60) than no surgery in the ITT analysis (Supplemental Table 6).

### Sensitivity analyses

Results did not change appreciably after excluding 6,915 (11.2%) individuals because of infertility, 1,535 (2.5%) because of <12 months of baseline data, or using IPCW (Supplemental Table 7).

When hysteroscopic myomectomy (CPT 58561) was separated from other minimally invasive myomectomy procedures, there was a reduction in atherosclerotic CVD risk compared with no surgery (ITT aRD −1.99%, 95% CI: −3.50, −0.28; Supplemental Table 8), whereas estimates for laparoscopic and robotic myomectomy remained directionally similar but less precise.

## DISCUSSION

Among individuals with uterine fibroids, there were imprecise associations between myomectomy and atherosclerotic CVD risk, while undergoing a hysterectomy (without oophorectomy) was associated with modestly increased atherosclerotic CVD risk.

Although confidence intervals around estimates for cardiovascular associations with myomectomy indicate uncertainty, the consistent direction of estimates may suggest potential cardiovascular benefits, highlighting the need for further research. This may be related to preservation of uterine and ovarian functions, with maintained cyclic hormonal patterns and menstrual function after surgery (58). Additionally, undergoing a myomectomy may provide benefits in preserving reproductive physiology while relieving fibroid-related symptoms, such as heavy bleeding, pelvic pressure, and anemia, that have been associated with increased cardiovascular strain (17). Interestingly, this does not seem to be driven by reductions in atherosclerotic CVD risk factors, in particular, incident type 2 diabetes, hypertension, hyperlipidemia, or obesity. Future research with more extended follow-up should aim to examine long-term cardiovascular associations after myomectomy.

The modestly increased risk of atherosclerotic CVD with hysterectomy is consistent with general reproductive cohorts, potentially explained by reduced ovarian blood flow when uterine arteries are divided (65). Alterations in cyclic hormonal patterns may have adverse effects on lipid metabolism, endothelial function, and vascular inflammation. (66, 67) Consistent with this, undergoing a hysterectomy was also associated with a higher 5-year risk of obesity, hypertension, and hyperlipidemia in this cohort, even after excluding individuals with these conditions at baseline. However, as these outcomes were ascertained via diagnosis codes, it is unclear whether they reflect truly incident conditions or residual differences between groups, including conditions that were present but previously undiagnosed.

Our findings extend prior hysterectomy and CVD studies (21, 51, 62, 63), which included postmenopausal cohorts, did not isolate uterine fibroid cases (24), and often did not distinguish procedures without oophorectomy, which have a less clear biological rationale for negative cardiovascular impact. A meta-analysis comparing hysterectomy with no surgery reported modest CVD and stroke risk increases (9%–10%) (23), whereas an analysis of the Nurses’ Health Study reported a 21% increased CVD risk, although in a cohort with an average age of 48 years (24). A cohort in Australia similarly found an increased risk associated with hysterectomy with ovarian conservation before 35 years (22). Only one study conducted a subgroup analysis exclusively among individuals with uterine fibroids (N=761), finding no difference in coronary artery disease or stroke risk after hysterectomy with ovarian conservation, despite harmful associations in their general cohort (20).

Our analyses stratified by route of surgery provide additional evidence. Open/abdominal hysterectomy was associated with a substantially higher 5-year atherosclerotic CVD risk compared with no surgery, whereas minimally invasive hysterectomy showed a modest and imprecise increase. This difference may reflect greater surgical trauma and tissue disruption with open procedures, or the selection of patients with more severe disease for open approaches. Hysterectomy with concurrent unilateral oophorectomy was associated with an even greater atherosclerotic CVD risk increase of 2.06%, consistent with the hypothesis that surgical disruption of ovarian function may be associated with cardiovascular risk through premature induction of a hypoestrogenic state. (21–23)

Several important limitations must be considered. Confounding by fibroid severity is an important concern and warrants careful consideration for each comparison. For myomectomy compared to no surgery, the theoretical direction of confounding by indication is reassuring, as individuals with more severe fibroids would be expected to have worse cardiovascular risk profiles, which would be expected to bias the myomectomy group toward a higher atherosclerotic CVD risk. Because myomectomy showed a modest protective association, this direction is inconsistent with what confounding by indication alone would produce and might suggest an underestimate. For hysterectomy, this argument does not apply in the same way, and residual confounding by disease severity cannot be excluded. Notably, open hysterectomy was associated with substantially greater atherosclerotic CVD risk than minimally invasive hysterectomy, a pattern that may reflect greater surgical trauma and vascular disruption, although differences in patient selection across surgical routes could also contribute. From a methodological standpoint, we attempted to reduce the potential for confounding by indication using IPTW with balanced observable proxies of disease severity, including fibroid-related symptoms, and we required imaging confirmation of diagnosis and an active fibroid claim within 1 month of each trial start.

Unmeasured aspects of fibroid severity, including size, number, and location, remain limitations of claims-based data, and residual confounding cannot be fully excluded. Additional limitations include reliance on billing codes for variable ascertainment and lack of data on surgical indications beyond fibroid diagnosis. Although we required a fibroid claim within 1 month of follow-up start to ensure active disease, hysterectomies may have been performed for cooccurring conditions such as endometriosis or uterine prolapse, which would need an independent causal relationship with atherosclerotic CVD to result in confounding. Additionally, the Optum CDM database population may limit generalizability to broader patient populations with uterine fibroids. Finally, the average length of follow-up in this cohort was shorter than expected, which limited power to detect differences in outcomes and could have led to an underestimation of risk differences, as atherosclerotic CVD events may develop over longer periods. Although our study was designed to emulate a target trial and estimate causal effects, the small number of events and resulting wide confidence intervals mean these findings should be interpreted as exploratory rather than definitive; caution is warranted in drawing strong causal conclusions from these estimates.

## CONCLUSION

Fibroid treatment decisions should be grounded in shared decision-making (58, 68–70). This study suggests that among individuals with uterine fibroids, myomectomy was not strongly associated with atherosclerotic CVD risk, whereas hysterectomy was associated with modest increases. These associations may inform patient counseling alongside established decision-making factors, yet further research is needed.

## Supplementary Material

1

2

Supplemental data for this article can be found online at https://doi.org/10.1016/j.fertnstert.2026.05.151.

## Figures and Tables

**FIGURE 1:**
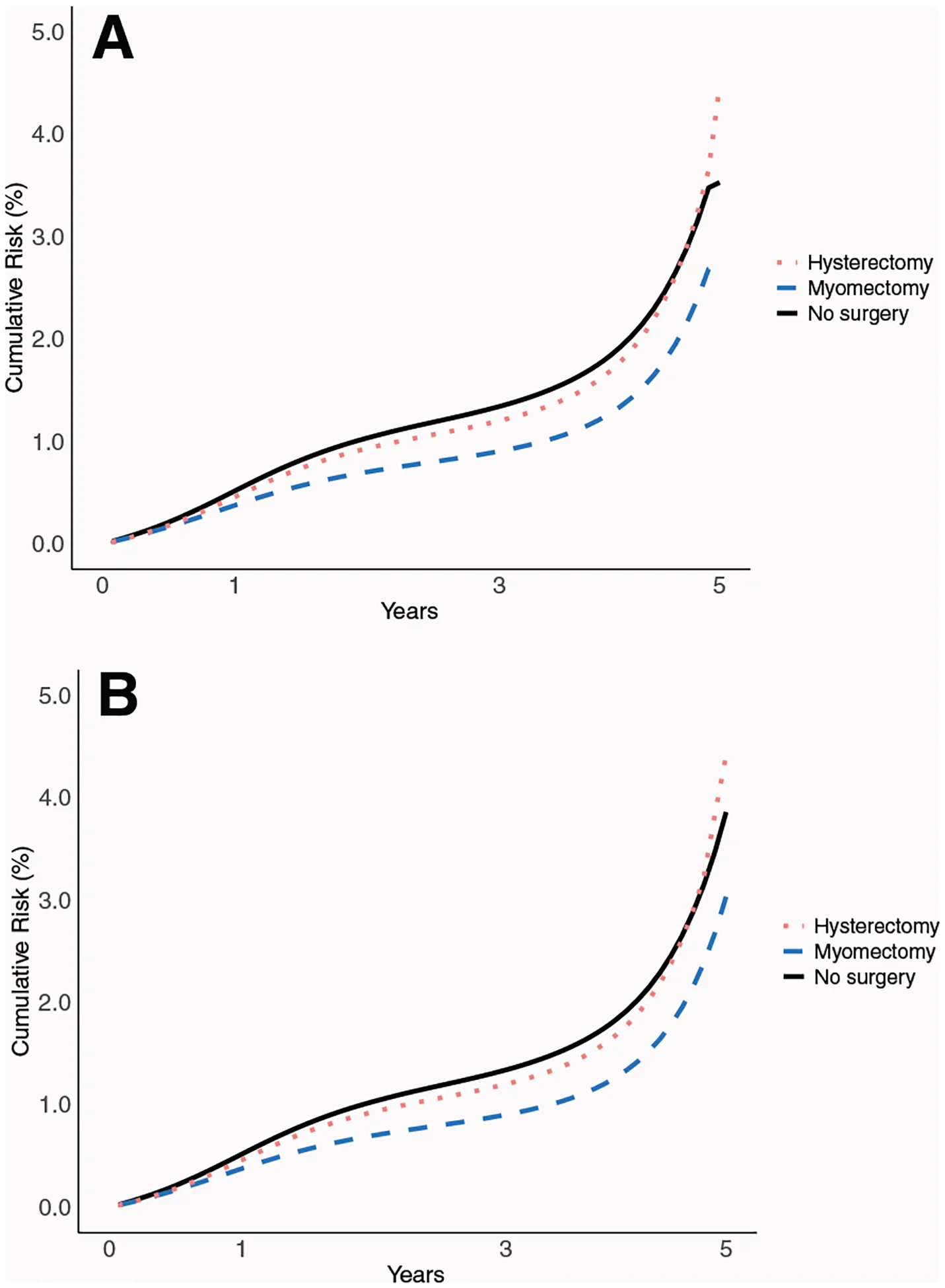
Intention-to-treat and per-protocol cumulative risk curves for atherosclerotic cardiovascular disease. (A) Intention-to-treat analysis.^a^ (B) Per-protocol analysis.^a,b a^Models included stabilized inverse probability of treatment weights estimated using the following covariates, measured at the baseline of each “trial:” age at uterine fibroid diagnosis, age at trial start, obesity, hyperlipidemia, type 2 diabetes mellitus (T2D), hypertension, smoking, obstructive sleep apnea (OSA), metabolic dysfunction-associated fatty liver disease (MAFLD), anxiety, depression, parity, infertility, uterine fibroid symptoms (pelvic pain, heavy menstrual bleeding), and prior medical management (oral and nonoral hormonal contraceptives). ^b^Per-protocol analysis: Models included time-varying stabilized inverse probability weights estimated using baseline and time-varying values of: age at uterine fibroid diagnosis, age at trial start, obesity, hyperlipidemia or use of statins, T2D or use of antidiabetic medications, hypertension or use of antihypertensives, smoking, OSA, MAFLD, parity, infertility or use of infertility medications, uterine fibroid symptoms, prior medical management.

**FIGURE 2:**
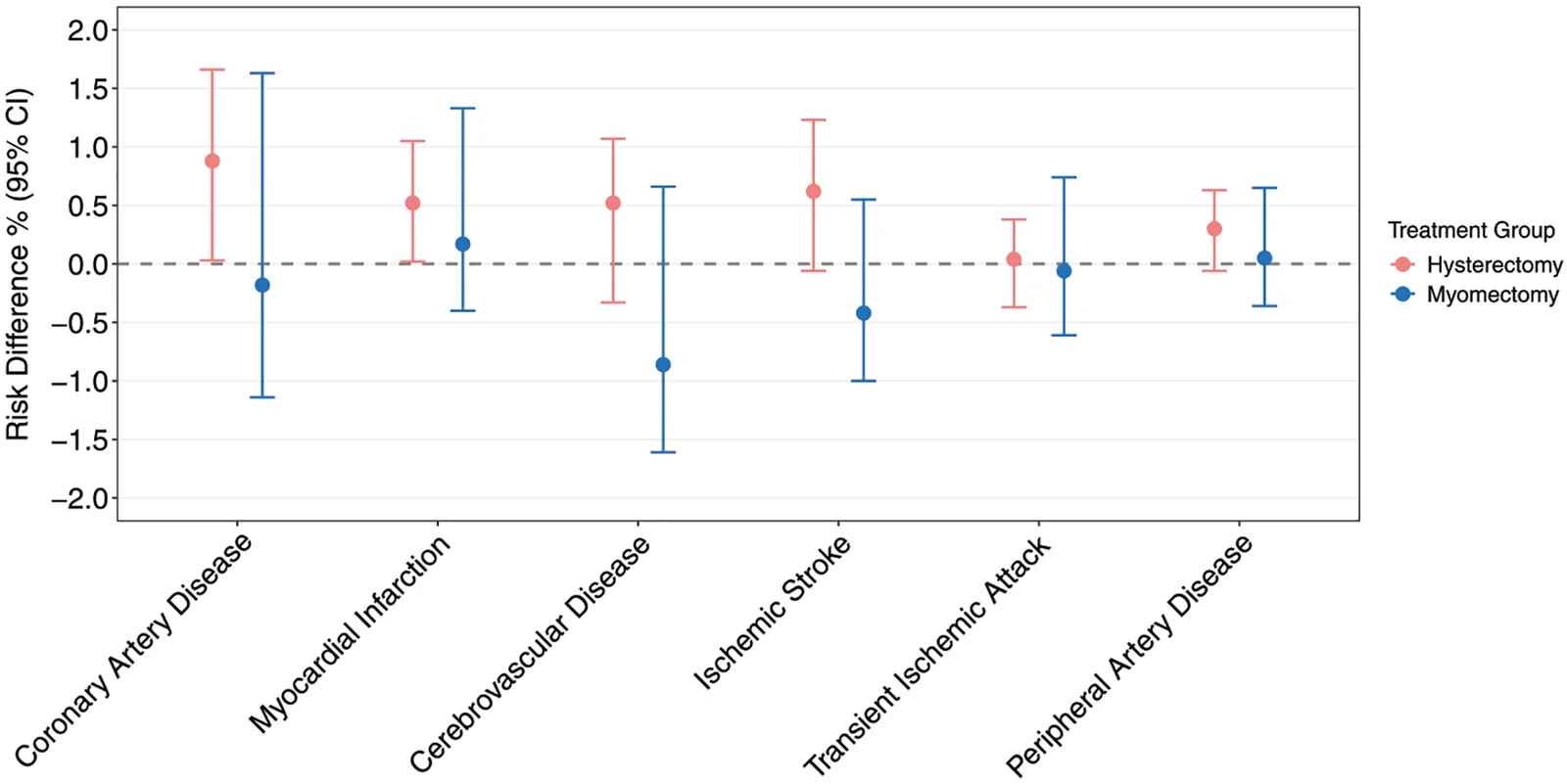
Five-year risk differences of individual atherosclerotic cardiovascular disease components and events by treatment group compared with no surgery. The reference group is no surgery. Confidence intervals of 95% were estimated using bootstrapping with 500 samples. Models included stabilized inverse probability of treatment weights estimated using the following covariates, measured at the baseline of each trial: age at uterine fibroid diagnosis, age at trial start, obesity, hyperlipidemia, Type 2 diabetes mellitus (T2D), hypertension, smoking, obstructive sleep apnea (OSA), metabolic dysfunction-associated fatty liver disease (MAFLD), anxiety, depression, parity, infertility, uterine fibroid symptoms (pelvic pain, heavy menstrual bleeding), and prior medical management (oral and nonoral hormonal contraceptives).

**TABLE 1:** Baseline characteristics of eligible individuals by treatment group, Optum CDM 2000–2024.

Baseline characteristic	Myomectomy	Hysterectomy	No surgery
No.	11,106	23,089	27,657
Demographic information			
Age at uterine fibroid diagnosis (y), median (IQR)	37.00 (32.00, 41.00)	41.00 (38.00, 44.00)	40.00 (36.00, 44.00)
Age at “trial” start (y), median (IQR)	37.00 (33.00, 42.00)	42.00 (38.00, 45.00)	41.00 (36.00, 45.00)
Clinical characteristics			
Obesity	1,059 (9.5)	3,199 (13.9)	3,269 (11.8)
Hyperlipidemia	1,900 (17.1)	5,241 (22.7)	6,243 (22.6)
Type 2 diabetes mellitus	463 (4.2)	1,451 (6.3)	1,693 (6.1)
Hypertension	1,239 (11.2)	4,653 (20.2)	4,862 (17.6)
Parity	1,138 (10.2)	2,764 (12.0)	3,154 (11.4)
Smoking	432 (3.9)	1,819 (7.9)	1,210 (4.4)
Anxiety	2,848 (25.6)	7,275 (31.5)	7,384 (26.7)
Depression	2,553 (23.0)	7,388 (32.0)	6,865 (24.8)
Infertility	1,524 (13.7)	489 (2.1)	1,562 (5.6)
Endometriosis	453 (4.1)	1,075 (4.7)	731 (2.6)
Uterine fibroid symptoms			
Any symptoms	7,546 (67.9)	17,542 (76.0)	17,946 (64.9)
Pelvic pain	5,082 (45.8)	11,964 (51.8)	12,850 (46.5)
Heavy menstrual bleeding	4,932 (44.4)	12,851 (55.7)	10,584 (38.3)
Medical management of uterine fibroids			
Hormonal contraceptive use	3,190 (28.7)	4,919 (21.3)	6,078 (22.0)
Tranexamic acid use	57 (0.5)	223 (1.0)	127 (0.5)
GnRH agonist use	90 (0.8)	42 (0.2)	106 (0.4)
Procedural management of uterine fibroids			
Uterine artery embolization	134 (1.2)	367 (1.6)	397 (1.4)
Endometrial ablation	88 (0.8)	853 (3.7)	412 (1.5)

*Note:* Data are presented as N (%), unless otherwise specified. IQR = interquartile range; GnRH = gonadotropin-releasing hormone.

**TABLE 2:** Five-year atherosclerotic cardiovascular disease risk estimates by treatment.

5-year atherosclerotic cardiovascular disease estimate	Myomectomy	Hysterectomy	No surgery
Events/person-months	149/331,816	506/681,341	606/836,940
Unadjusted analysis^a^			
RD (95% CI)	−1.90% (−2.98, −0.96)	1.74% (0.82, 2.64)	(ref)
RR (95% CI)	0.70 (0.55, 0.84)	1.28 (1.13, 1.45)	(ref)
Intention-to-treat analysis^a,b^			
aRD (95% CI)	−0.93% (−2.04, 1.30)	1.24% (−0.01, 2.03)	(ref)
aRR (95% CI)	0.85 (0.68, 1.22)	1.20 (1.00, 1.34)	(ref)
Per-protocol analysis^a,b,c^			
aRD (95% CI)	−0.98% (−2.10, 1.27)	1.39% (0.02, 2.16)	(ref)
aRR (95% CI)	0.84 (0.66, 1.21)	1.23 (1.00, 1.38)	(ref)

*Note:*.

a 95% confidence intervals are estimated using bootstrapping with 500 samples.

b Models included stabilized inverse probability of treatment weights estimated using the following covariates, measured at the baseline of each “trial:” age at uterine fibroid diagnosis, age at “trial” start, obesity, hyperlipidemia, Type 2 diabetes mellitus (T2D), hypertension, smoking, obstructive sleep apnea (OSA), metabolic dysfunction-associated fatty liver disease (MAFLD), anxiety, depression, parity, infertility, uterine fibroid symptoms (pelvic pain, heavy menstrual bleeding), and prior medical management (oral and nonoral hormonal contraceptives).

c Per-protocol analysis: Models included time-varying stabilized inverse probability weights estimated using baseline and time-varying values of: age at uterine fibroid diagnosis, age at “trial” start, obesity, hyperlipidemia or use of statins, T2D or use of antidiabetic medications, hypertension or use of antihypertensives, smoking, OSA, MAFLD, parity, infertility or use of infertility medications, uterine fibroid symptoms, prior medical management. aRD = adjusted risk difference; aRR = adjusted risk ratio;; CI = confidence interval; RD = risk difference; ref = reference; RR = risk ratio; T2D = type 2 diabetes mellitus.

## References

[1] Baird DD, Dunson DB, Hill MC, Cousins D, Schectman JM. High cumulative incidence of uterine leiomyoma in black and white women: ultrasound evidence. Am J Obstet Gynecol 2003;188:100–7. 10.1067/mob.2003.99 12548202

[2] Eltoukhi HM, Modi MN, Weston M, Armstrong AY, Stewart EA. The health disparities of uterine fibroid tumors for African American women: a public health issue. Am J Obstet Gynecol 2014;210:194–9. 10.1016/j.ajog.2013.08.008 PMC3874080 23942040

[3] Arnett DK, Blumenthal RS, Albert MA, Buroker AB, Goldberger ZD, Hahn EJ, 2019 ACC/AHA guideline on the primary prevention of cardiovascular disease: executive summary: a report of the American College of Cardiology/American Heart Association Task Force on Clinical Practice Guidelines. Circulation 2019;140:e563–95. 10.1161/CIR.0000000000000677 PMC8351755 30879339

[4] Ahmad FB, Cisewski JA, Anderson RN. Report No. 39 Mortality in the United States: provisional data, 2024. In: Vital statistics rapid release reports [Internet]. Hyattsville, MD: National Center for Health Statistics; 2025.

[5] Alnefaie K, Dungan JR. Reproductive-associated risk factors and incident coronary heart disease in women: an umbrella review. Am Heart J Plus 2025;55:100558. 10.1016/j.ahjo.2025.100558 PMC12162073 40510548

[6] Colombo GE, Mahamat-Saleh Y, Armour M, Madan K, Sabag A, Kvaskoff M, Non-malignant gynaecological disease and risk of cardiovascular or cerebrovascular disease: a systematic review and meta-analysis. Heart 2025;111:402–11. 10.1136/heartjnl-2024-324675 39993911

[7] Tripathi A, Arsha S, Thapa A, Thapa S, Chand S, Frishman WH, Cardiovascular implications of gynecological disorders: bridging the gap between gynecology and cardiology. Cardiol Rev 2024, in press. 10.1097/CRD.0000000000000759 39078163

[8] DiTosto JD, Lewey J, Zee J, Dokras A, Busse KR, Alur-Gupta S, Association between uterine fibroids and risk of atherosclerotic cardiovascular disease. J Am Heart Assoc 2026;15:e044014. 10.1161/JAHA.125.044014 PMC12909019 41371752

[9] Brewster LM, Haan Y, van Montfrans GA. Cardiometabolic risk and cardiovascular disease in young women with uterine fibroids. Cureus 2022;14:e30740. 10.7759/cureus.30740 PMC9699995 36447683

[10] Chen Y, Xiong N, Xiao J, Huang X, Chen R, Ye S, Association of uterine fibroids with increased blood pressure: a cross-sectional study and meta-analysis. Hypertens Res 2022;45:715–21. 10.1038/s41440-022-00856-w 35169279

[11] Laughlin-Tommaso SK, Fuchs EL, Wellons MF, Lewis CE, Calderon-Margalit R, Stewart EA, Uterine fibroids and the risk of cardiovascular disease in the coronary artery risk development in young adult women’s study. J Womens Health (Larchmt) 2019;28:46–52. 10.1089/jwh.2018.7122 PMC6343187 30412447

[12] Kirschen GW, AlAshqar A, Miyashita-Ishiwata M, Reschke L, El Sabeh M, Borahay MA. Vascular biology of uterine fibroids: connecting fibroids and vascular disorders. Reproduction 2021;162:R1–18. 10.1530/REP-21-0087 PMC8320308 34034234

[13] AlAshqar A, Patzkowsky K, Afrin S, Wild R, Taylor HS, Borahay MA. Cardiometabolic risk factors and benign gynecologic disorders. Obstet Gynecol Surv 2019;74:661–73. 10.1097/OGX.0000000000000718 PMC6986276 31755543

[14] Moravek MB, Bulun SE. Endocrinology of uterine fibroids: steroid hormones, stem cells, and genetic contribution. Curr Opin Obstet Gynecol 2015;27:276–83. 10.1097/GCO.0000000000000185 PMC4734398 26107781

[15] Merrill RM. Hysterectomy surveillance in the United States, 1997 through 2005. Med Sci Monit 2008;14:Cr24–31. 18160941

[16] Wright JD, Herzog TJ, Tsui J, Ananth CV, Lewin SN, Lu YS, Nationwide trends in the performance of inpatient hysterectomy in the United States. Obstet Gynecol 2013;122(Pt 1):233–41. 10.1097/AOG.0b013e318299a6cf PMC3913114 23969789

[17] Anchan RM, Spies JB, Zhang S, Wojdyla D, Bortoletto P, Terry K, Long-term health-related quality of life and symptom severity following hysterectomy, myomectomy, or uterine artery embolization for the treatment of symptomatic uterine fibroids. Am J Obstet Gynecol 2023;229:275.e1, 17. 10.1016/j.ajog.2023.05.020 37244458

[18] Harvey SV, Pfeiffer RM, Landy R, Wentzensen N, Clarke MA. Trends and predictors of hysterectomy prevalence among women in the United States. Am J Obstet Gynecol 2022;227(611):e1–12. 10.1016/j.ajog.2022.06.028 PMC9529796 35764133

[19] Borah BJ, Yao X, Laughlin-Tommaso SK, Heien HC, Stewart EA. Comparative effectiveness of uterine leiomyoma procedures using a large insurance claims database. Obstet Gynecol 2017;130:1047–56. 10.1097/AOG.0000000000002331 PMC5683097 29016510

[20] Laughlin-Tommaso SK, Khan Z, Weaver AL, Schleck CD, Rocca WA, Stewart EA. Cardiovascular risk factors and diseases in women undergoing hysterectomy with ovarian conservation. Menopause 2016;23:121–8. 10.1097/GME.0000000000000506 PMC4712122 26173076

[21] Yuk JS, Kim BG, Lee BK, Seo J, Kim GS, Min K, Association of early hysterectomy with risk of cardiovascular disease in Korean women. J Am Med Assoc Netw Open 2023;6:e2317145. 10.1001/jamanetworkopen.2023.17145 PMC10261994 37307002

[22] Tuesley KM, Protani MM, Webb PM, Dixon-Suen SC, Wilson LF, Stewart LM, Hysterectomy with and without oophorectomy and all-cause and cause-specific mortality. Am J Obstet Gynecol 2020;223:723.e1, 16. 10.1016/j.ajog.2020.04.037 32376318

[23] Chen Y, Li F, Liang L, Hua H, Liu S, Yu Z, Examining the association of hysterectomy with and without oophorectomy on cardiovascular disease and all-cause, cardiovascular or cancer mortality: a systematic review and meta-analysis. Br J Obstet Gynaecol 2024;131:1444–55. 10.1111/1471-0528.17843 38747094

[24] Thao V, Borah B, Stewart EA, Farland LV, Laughlin-Tommaso SK, Khan Z, Cardiovascular disease after hysterectomy in the Nurses’ Health Study and Nurses’ Health Study II. Obstet Gynecol 2025;146:85–93. 10.1097/AOG.0000000000005902 PMC12221304 40341551

[25] Matthews KA, Gibson CJ, El Khoudary SR, Thurston RC. Changes in cardiovascular risk factors by hysterectomy status with and without oophorectomy: study of women’s health across the nation. J Am Coll Cardiol 2013;62:191–200. 10.1016/j.jacc.2013.04.042 PMC3777736 23684687

[26] Howard BV, Kuller L, Langer R, Manson JE, Allen C, Assaf A, Risk of cardiovascular disease by hysterectomy status, with and without oophorectomy: the women’s health initiative observational study. Circulation 2005;111:1462–70. 10.1161/01.CIR.0000159344.21672.FD 15781742

[27] Kharazmi E, Fallah M, Luoto R. Cardiovascular diseases attributable to hysterectomy: a population-based study. Acta Obstet Gynecol Scand 2007;86:1476–83. 10.1080/00016340701698633 17917816

[28] Matthews KA, Crawford SL, Chae CU, Everson-Rose SA, Sowers MF, Sternfeld B, Are changes in cardiovascular disease risk factors in midlife women due to chronological aging or to the menopausal transition? J Am Coll Cardiol 2009;54:2366–73. 10.1016/j.jacc.2009.10.009 PMC2856606 20082925

[29] Iyer TK, Manson JE. Hysterectomy with or without ovarian conservation: similar associations with vascular health? Menopause 2023;30:687–9. 10.1097/GME.0000000000002208 PMC10330145 37339392

[30] Bonine NG, Banks E, Harrington A, Vlahiotis A, Moore-Schiltz L, Gillard P. Contemporary treatment utilization among women diagnosed with symptomatic uterine fibroids in the United States. BMC Womens Health 2020;20:174. 10.1186/s12905-020-01005-6 PMC7427077 32791970

[31] El Khoudary SR, Thurston RC. Cardiovascular implications of the menopause transition: endogenous sex hormones and vasomotor symptoms. Obstet Gynecol Clin North Am 2018;45:641–61. 10.1016/j.ogc.2018.07.006 30401548

[32] Huang Y, Wu M, Wu C, Zhu Q, Wu T, Zhu X, Effect of hysterectomy on ovarian function: a systematic review and meta-analysis. J Ovarian Res 2023;16:35. 10.1186/s13048-023-01117-1 PMC9912518 36759829

[33] Caniglia EC, Zash R, Fennell C, Diseko M, Mayondi G, Heintz J, Emulating target trials to avoid immortal time bias—an application to antibiotic initiation and preterm delivery. Epidemiology 2023;34:430–8. 10.1097/EDE.0000000000001601 PMC10263190 36805380

[34] Danaei G, García Rodríguez LA, Cantero OF, Logan RW, Hernán MA. Electronic medical records can be used to emulate target trials of sustained treatment strategies. J Clin Epidemiol 2018;96:12–22. 10.1016/j.jclinepi.2017.11.021 PMC5847447 29203418

[35] Kuehne F, Arvandi M, Hess LM, Faries DE, Matteucci Gothe R, Gothe H, Causal analyses with target trial emulation for real-world evidence removed large self-inflicted biases: systematic bias assessment of ovarian cancer treatment effectiveness. J Clin Epidemiol 2022;152:269–80. 10.1016/j.jclinepi.2022.10.005 36252741

[36] Hernán MA, Robins JM. Using big data to emulate a target trial when a randomized trial is not available. Am J Epidemiol 2016;183:758–64. 10.1093/aje/kwv254 PMC4832051 26994063

[37] Hernán MA, Sauer BC, Hernández-Díaz S, Platt R, Shrier I. Specifying a target trial prevents immortal time bias and other self-inflicted injuries in observational analyses. J Clin Epidemiol 2016;79:70–5. 10.1016/j.jclinepi.2016.04.014 PMC5124536 27237061

[38] Hernán MA, Wang W, Leaf DE. Target trial emulation: a framework for causal inference from observational data. J Am Med Assoc 2022;328:2446–7. 10.1001/jama.2022.21383 36508210

[39] Caniglia EC, Rojas-Saunero LP, Hilal S, Licher S, Logan R, Stricker B, Emulating a target trial of statin use and risk of dementia using cohort data. Neurology 2020;95:e1322–32. 10.1212/WNL.0000000000010433 PMC7538212 32753444

[40] Matthews AA, Szummer K, Dahabreh IJ, Lindahl B, Erlinge D, Feychting M, Comparing effect estimates in randomized trials and observational studies from the same population: an application to percutaneous coronary intervention. J Am Heart Assoc 2021;10:e020357. 10.1161/JAHA.120.020357 PMC8483524 33998290

[41] Cashin AG, Hansford HJ, Hernán MA, Swanson SA, Lee H, Jones MD, Transparent reporting of observational studies emulating a target trial: the TARGET Statement. Br Med J 2025;390:e087179. 10.1136/bmj-2025-087179 40903028

[42] Chiuve SE, Huisingh C, Petruski-Ivleva N, Owens C, Kuohung W, Wise LA. Uterine fibroids and incidence of depression, anxiety and self-directed violence: a cohort study. J Epidemiol Community Health 2022;76:92–9. 10.1136/jech-2020-214565 PMC8666805 34301795

[43] Charifson M, Beaton-Mata G, Lipschultz R, Robinson I, Sasse SA, Hur HC, Using electronic health record data to identify incident uterine fibroids and endometriosis within a large, urban academic medical center: a validation study. Am J Epidemiol 2026;195:291–9. 10.1093/aje/kwaf058 PMC13368599 40102190

[44] Jacoby VL, Sawaya G. The swinging pendulum: elective oophorectomy at the time of hysterectomy. Obstet Gynecol 2022;139:721–3. 10.1097/AOG.0000000000004776 35576329

[45] Danaei G, Rodríguez LA, Cantero OF, Logan R, Hernán MA. Observational data for comparative effectiveness research: an emulation of randomised trials of statins and primary prevention of coronary heart disease. Stat Methods Med Res 2013;22:70–96. 10.1177/0962280211403603 PMC3613145 22016461

[46] García-Albéniz X, Hsu J, Bretthauer M, Hernán MA. Effectiveness of screening colonoscopy to prevent colorectal cancer among medicare beneficiaries aged 70 to 79 years: a prospective observational study. Ann Intern Med 2017;166:18–26. 10.7326/M16-0758 PMC5417337 27669524

[47] Hernán MA, Alonso A, Logan R, Grodstein F, Michels KB, Willett WC, Observational studies analyzed like randomized experiments: an application to postmenopausal hormone therapy and coronary heart disease. Epidemiology 2008;19:766–79. 10.1097/EDE.0b013e3181875e61 PMC3731075 18854702

[48] Stampfer MJ. ITT for observational data: worst of both worlds? Epidemiology 2008;19(6):783–4 [discussion: 9–93]. 10.1097/EDE.0b013e318188442e 18813017

[49] Davis MR, Soliman AM, Castelli-Haley J, Snabes MC, Surrey ES. Reintervention rates after myomectomy, endometrial ablation, and uterine artery embolization for patients with uterine fibroids. J Womens Health (Larchmt) 2018;27:1204–14. 10.1089/jwh.2017.6752 PMC6205049 30085898

[50] Ding DC, Tsai IJ, Wang JH, Lin SZ, Sung FC. Coronary artery disease risk in young women with polycystic ovary syndrome. Oncotarget 2018;9:8756–64. 10.18632/oncotarget.23985 PMC5823557 29492235

[51] Ding DC, Tsai IJ, Hsu CY, Wang JH, Lin SZ. Hysterectomy is associated with higher risk of coronary artery disease: a nationwide retrospective cohort study in Taiwan. Medicine (Baltimore) 2018;97:e0421. 10.1097/MD.0000000000010421 PMC5916687 29668602

[52] Gallardo-Gómez D, Dwan K. Standardised mean difference. Br Med J Evid Based Med 2026;31:132–4. 10.1136/bmjebm-2024-113579 40441872

[53] D’Agostino RB, Lee ML, Belanger AJ, Cupples LA, Anderson K, Kannel WB. Relation of pooled logistic regression to time dependent Cox regression analysis: the Framingham heart study. Stat Med 1990;9:1501–15. 10.1002/sim.4780091214 2281238

[54] Katon JG, Plowden TC, Marsh EE. Racial disparities in uterine fibroids and endometriosis: a systematic review and application of social, structural, and political context. Fertil Steril 2023;119:355–63. 10.1016/j.fertnstert.2023.01.022 PMC9992263 36682686

[55] Grant JK, Martin SS, Zhang S, Matsushita K, Virani SS, Blumenthal RS, Racial differences in the burden of atherosclerotic cardiovascular disease related to elevated lipoprotein(a) levels: the ARIC Study. Circulation 2024;150:250–2. 10.1161/CIRCULATIONAHA.124.069582 39008561

[56] Prior JC, Hitchcock CL. The endocrinology of perimenopause: need for a paradigm shift. Front Biosci (Schol Ed) 2011;3:474–86. 10.2741/s166 21196391

[57] Appiah D, Nwabuo CC, Ebong IA, Wellons MF, Winters SJ. Trends in age at natural menopause and reproductive life span among US women, 1959–2018. J Am Med Assoc 2021;325:1328–30. 10.1001/jama.2021.0278 PMC8025101 33821908

[58] Management of symptomatic uterine leiomyomas. ACOG practice bulletin, number 228. Obstet Gynecol 2021;137:e100–15. 10.1097/AOG.0000000000004401 34011888

[59] Laughlin-Tommaso SK, Lu D, Thomas L, Diamond MP, Wallace K, Wegienka G, Short-term quality of life after myomectomy for uterine fibroids from the COMPARE-UF fibroid registry. Am J Obstet Gynecol 2020;222:345.e1, 22. 10.1016/j.ajog.2019.09.052 31678093

[60] Nicholson WK, Wegienka G, Zhang S, Wallace K, Stewart E, Laughlin-Tommaso S, Short-term health-related quality of life after hysterectomy compared with myomectomy for symptomatic leiomyomas. Obstet Gynecol 2019;134:261–9. 10.1097/AOG.0000000000003354 PMC8579313 31306318

[61] Farland LV, Rice MS, Degnan WJ 3rd, Rexrode KM, Manson JE, Rimm EB, Hysterectomy with and without oophorectomy, tubal ligation, and risk of cardiovascular disease in the nurses’ health study II. J Womens Health (Larchmt) 2023;32:747–56. 10.1089/jwh.2022.0207 PMC10354306 37155739

[62] Parker WH, Broder MS, Chang E, Feskanich D, Farquhar C, Liu Z, Ovarian conservation at the time of hysterectomy and long-term health outcomes in the nurses’ health study. Obstet Gynecol 2009;113:1027–37. 10.1097/AOG.0b013e3181a11c64 PMC3791619 19384117

[63] Parker WH, Feskanich D, Broder MS, Chang E, Shoupe D, Farquhar CM, Long-term mortality associated with oophorectomy compared with ovarian conservation in the nurses’ health study. Obstet Gynecol 2013;121:709–16. 10.1097/AOG.0b013e3182864350 PMC4254662 23635669

[64] Mikhail E, Salemi JL, Mogos MF, Hart S, Salihu HM, Imudia AN. National trends of adnexal surgeries at the time of hysterectomy for benign indication, United States, 1998–2011. Am J Obstet Gynecol 2015;213:713.e1, 13. 10.1016/j.ajog.2015.04.031 25935772

[65] Moorman PG, Myers ER, Schildkraut JM, Iversen ES, Wang F, Warren N. Effect of hysterectomy with ovarian preservation on ovarian function. Obstet Gynecol 2011;118:1271–9. 10.1097/AOG.0b013e318236fd12 PMC3223258 22067716

[66] Turner CG, DuPont JJ. The effect of transient sex hormone fluctuations on vascular endothelial function. Am J Physiol Heart Circ Physiol 2025;329:H217–32. 10.1152/ajpheart.00174.2025 PMC12213119 40459955

[67] van Oortmerssen JAE, Mulder JWCM, Kavousi M, Roeters van Lennep JE. Lipid metabolism in women: a review. Atherosclerosis 2025;405:119213. 10.1016/j.atherosclerosis.2025.119213 40300433

[68] Laughlin SK, Stewart EA. Uterine leiomyomas: individualizing the approach to a heterogeneous condition. Obstet Gynecol 2011;117(Pt 1):396–403. 10.1097/AOG.0b013e31820780e3 PMC3150866 21252757

[69] Wolgemuth T, Suresh N, Holder K, Milad MP. Informational preferences of women seeking treatment for symptomatic uterine fibroids: a qualitative study. J Minim Invasive Gynecol 2023;30:284–9. 10.1016/j.jmig.2022.12.008 36528257

[70] Templeman C, Marshall SF, Clarke CA, DeLellis Henderson K, Largent J, Neuhausen S, Risk factors for surgically removed fibroids in a large cohort of teachers. Fertil Steril 2009;92:1436–46. 10.1016/j.fertnstert.2008.08.074 PMC2765807 19019355

