# Supplementary material for: Emulating a target trial of surgical removal of uterine fibroids on atherosclerotic cardiovascular disease
Source: Fertil Steril. Author manuscript; Available in PMC 2026 Oct 2. (PMC13631833; doi:10.1016/j.fertnstert.2026.05.151)
Supplement: Supplementary file 1 [file NIHMS2212450-supplement-1.docx]

Table of Contents

[Supplemental Table 1. ICD-9 and 10, CPT/HCPCS, and NDC/AHFS Classification Codes for Eligibility Criteria, Exposure, Outcomes, and Covariates.	2](#_Toc227845103)

[Supplemental Table 2. Target Trial Emulation Protocol.	7](#_Toc227845104)

[Supplemental Figure 1. Schematic Representation of Sequential Target Trial Emulation.	9](#_Toc227845105)

[Supplemental Figure 2. Study Population and Cohort Enrollment Process, Optum CDM® 2000-2019.	10](#_Toc227845106)

[Supplemental Figure 3. Standardized Mean Differences of Baseline Characteristics Before and After Weighting.	11](#_Toc227845107)

[Supplemental Table 3. 5-Year Regression Results for Secondary Outcomes of Individual Atherosclerotic Cardiovascular Disease Events, Diagnoses, and Risk Factors.	12](#_Toc227845108)

[Supplemental Table 4. Subgroup Analyses by Self-Reported Race and Ethnicity.	14](#_Toc227845109)

[Supplemental Table 5. Subgroup Analyses by Age at Trial Start.	15](#_Toc227845110)

[Supplemental Table 6. 5-Year Regression Results for Exploratory Analyses	16](#_Toc227845111)

[Supplemental Table 7. Sensitivity Analyses for Atherosclerotic Cardiovascular Disease Risk.	17](#_Toc227845112)

[Supplemental Table 8. 5-Year Regression Results for Sensitivity Analyses of Surgical Route	18](#_Toc227845113)

# **Supplemental Table 1. ICD-9 and 10, CPT/HCPCS, and NDC/AHFS Classification Codes for Eligibility Criteria, Exposure, Outcomes, and Covariates.**

| **Variable** | **ICD-9**  **(Diagnosis & Procedure)** | **ICD-10**  **(Diagnosis & Procedure)** | **CPT/HCPCS** | **NDC/AHFS Classification Codes** |
| --- | --- | --- | --- | --- |
| **Treatment groups** | | | | |
| Hysterectomy |  |  |  |  |
| Open/abdominal | 683.9, 684.9, 686.9 | 0UT90ZZ, 0UT90ZL | 58150, 58152, 58180, 58200, 58210, 58240 |  |
| Minimally invasive | 683.1, 684.1, 685.1, 685.9, 686.1, 687.1, 687.9 | 0UT94ZZ, 0UT97ZZ, 0UT98ZZ, 0UT9FZZ, 0UT94ZL, 0UT97ZL, 0UT98ZL, 0UT9FZL | 58260, 58262, 58263, 58267, 58270, 58275, 58280, 58285, 58290-58294, 58541-58544, 58548, 58550, 58552-58554, 58570-58573 |  |
| Route not specified | 689 | 0UT98ZZ |  |  |
| Myomectomy |  |  |  |  |
| Open/abdominal |  | 0UB90ZZ | 58140, 58146 |  |
| Minimally invasive |  | 0UB94ZZ, 0UB97ZZ, 0UB98ZZ | 58145, 58545, 58546, 58561 |  |
| Route not specified | 682.9 |  |  |  |
| Unilateral oophorectomy | 653, 653.1, 653.9, 654.1, 654.9 | 0UT00ZZ, 0UT04ZZ, 0UT10ZZ, 0UT14ZZ | 58720, 58940, 58943 |  |
| **Eligibility criteria** | | | | |
| Uterine fibroids | 218.X | D25.X |  |  |
| Pelvic MRI and transvaginal ultrasonography | 890 | 8E0WXBH, BR3CY0Z, BR3CYZZ, BR3CZZZ, BR3FY0Z, BR3FYZZ, BR3FZZZ, BW3GY0Z, BW3GYZZ, BW3GZZZ | 72195-72197, 74181-74183, 76830, 76856 |  |
| Oophorectomy | 653-656.X | 0UT00ZZ, 0UT04ZZ, 0UT10ZZ, 0UT14ZZ, 0UT20ZZ, 0UT24ZZ | 58661, 58720, 58940, 58943 |  |
| Menopause | 627.1-627.4, 627.8, 627.9, 256.31 | N95.0-N95.2, N95.8, N95.9 |  |  |
| Cancer | 140.0-209.79 | C00.0-C96.Z |  |  |
| **Outcomes** | | | | |
| Coronary artery disease | 410.X-414.X, 427.5, 36.04, 36.06, 36.07, 36.09, 36.1 | I20.X-I25.X, I46.2, I46.8, I46.9, 027.0-027.3, 021.0, 021.2, 021.3 | 33510-33536, 92920-92996 |  |
| Myocardial infarction | 410.X-412 | I21.X-I23.8, I46.2 |  |  |
| Cerebrovascular disease | 433.01-438.9, 362.31-363.34, 435.X-438.X, 674.X, 0.63, 38.12 | G45.X-G46.8, I63.X-I69.398, H34.X, 03CH0-03CL3, 037H3-037L3 | 37215, 37216, 35301 |  |
| Transient ischemic attack | 435.X | G45.1-G45.9, I63.00 |  |  |
| Ischemic stroke | 433.01-438.X, 362.31-363.34 | I63.X-I70.201 |  |  |
| Peripheral artery disease | 440.2-440.9, 38.08-39.9 | I70.201-I70.92, F32.0, 041C-047Y, 04CC-04CY | 0238T, 35381-37235 |  |
| **Clinical variables** | | | | |
| Obesity | 278.00-278.03, 649.1X | E66.01-E66.9, O99.21X |  |  |
| Hyperlipidemia or statin use | 272.0-272.9 | E78.0-E78.6 |  | AHFS Class 240608% |
| Type 2 diabetes or diabetes medication use | 249.00-250.93, 357.2, 366.41, 362.01-362.07 | E08.00-E13.9, O24.011-O24.93 |  | AHFS Class 682008%, 68200600, 68202000; Generic: METFORMIN HCL, METFORMIN/AA 7/HERB125/CHOLINE, METFORMIN/BLOOD SUGAR DIAGNOST, METFORMIN/CAFF/AA7/HRB125/CHOL |
| Hypertension or antihypertensive use | 401.0-404.93, 642.00-642.94 | I10-I13.2, O10.011-O11.9 |  | AHFS Class 2408% |
| Smoking | 305.1, 649.0X | F17.2XX, O99.33X |  |  |
| Anxiety or use of anxiolytics | 293.84, 300.0X, 309.21, 309.24, 309.28, 309.8X | F41.X, F43.22, F43.23 |  | AHFS Class 2824% |
| Depression or use of antidepressants | 296.2X-296.3X, 298.0, 309.0, 309.1, 309.28, 311 | F32.1-F34.1, F41.0 |  | AHFS Class 281604% |
| Parity | V27.X, V30, V31, V34, V36, V39, 646.X, 656.4X, 69.02, 69.52, 72.X-74.X, 644.2X, 765.X | Z37.X-Z38.8, O31.X, P95, O36.4XX, O60.1XX, P07.2X-P07.3X, O24.4X, O14.XX, 0U7C0D-0UQMXZZ, 0W8NXZZ, 10D17ZZ-10S0XZZ | 00850-99465 |  |
| Infertility or use of infertility medications (including uterine factor infertility) | 628.X, V26.X | N97.X, Z31.X |  | AHFS Class 68180800; Generic: CLOMIPHENE CITRATE |
| Endometriosis | 617.X | N80.X |  |  |
| **Fibroid-related variables** | | | | |
| Pelvic pain | 625.3, 625.0, 302.67, 625.8, 625.9, 789.0X | N94.1X, N94.89, R10.1X-R10.84, R10.9 |  |  |
| Heavy menstrual bleeding | 280.X-281.9, 999.6X-999.89, 626.2, 626.8, 626.9, 627.0 | D50.X-D53.9, T80.3XX-T80.92X, Y65.0, N92.0, N92.1, N92.4 |  |  |
| Hormonal contraceptive use (Hormonal IUD, Vaginal Ring, Depo-Provera, Patch, Implant, Oral Contraceptive Pills) | V25.1, V25.11 | Z30.430, Z30.014 | 58300, J7301, J7302, S4981, J7296-J7298, Q0090, S4980, J7306, J7307, 11983, J1050, J1051, J1055 | NDC: 50419042101, 50419042301, 50419042308, 50419042401, 50419042408, 00023585801, 52544003554, 50419042201, 00052027201, 00052027401, 00052433001, 78206014501, 00008256401; Generic: ESTRAD CYP/M-PROGEST ACET, MEDROXYPROGESTERONE AC/MICRO, ETONOGESTREL%, ETONOGESTREL/ETHINYL ESTRADIOL%, NORELGESTROMIN/ETHIN.ESTRADIOL%, DESOG-E.ESTRADIOL/E.ESTRADIOL, DESOGESTREL-ETHINYL ESTRADIOL, DROSPIR/ETH ESTRA/LEVOMEFOL CA, DROSPIRENONE, DROSPIRENONE/ESTETROL, ESTRADIOL VALERATE/DIENOGEST, ETHINYL ESTRADIOL/DROSPIRENONE, ETHYNODIOL D-ETHINYL ESTRADIOL, L-NORGEST-ETH ESTR/PRG TST KIT, L-NORGEST/E.ESTRADIOL-E.ESTRAD, LEVONORGEST/ETH.ESTRADIOL/IRON, LEVONORGESTREL/ETHIN.ESTRADIOL, NORETH-ETHINYL ESTRADIOL/IRON, NORETHINDRONE, NORETHINDRONE AC-ETH ESTRADIOL, NORETHINDRONE-E.ESTRADIOL-IRON, NORETHINDRONE-ETHIN. ESTRADIOL, NORETHINDRONE-MESTRANOL, NORGESTIMATE-ETHINYL ESTRADIOL, NORGESTREL, NORGESTREL-ETHINYL ESTRADIOL, ULIPRISTAL ACETATE, NORETHINDRONE ACETATE |
| Tranexamic acid use |  |  |  | Generic: TRANEXAMIC ACID% |
| GnRH agonist use |  |  |  | Generic: GONADORELIN ACETATE, GONADORELIN HCL, GOSERELIN ACETATE, HISTRELIN ACETATE, LEUPROLIDE ACETATE, LEUPROLIDE ACETATE/LIDOCAINE, LEUPROLIDE MESYLATE, LEUPROLIDE/NORETHINDRONE ACET, NAFARELIN ACETATE, TRIPTORELIN PAMOATE |
| Uterine artery embolization | 682.4, 682.5, 388, 397.9, 992.9 | 04LE0XX-04LF4ZU | 37204, 37210, 37243 |  |
| Endometrial ablation | 682.3, 683.4, 683.9 | 0U5B0ZZ-0UDB8ZZ | 58353, 58356, 58563, 0071T, 0072T, 49203-49205 |  |

# **Supplemental Table 2. Target Trial Emulation Protocol.**

|  | **Target trial protocol** | **Emulation using Optum CDM®** |
| --- | --- | --- |
| **Eligibility criteria** | Females aged 18-50 years with new or existing diagnosis of uterine fibroids and no history of atherosclerotic CVD, myomectomy, hysterectomy, oophorectomy (bilateral or unilateral), menopause, or cancer at the time of enrollment in the study  Individuals who are pregnant or within 3 months postpartum and those who undergo a hysterectomy with concurrent oophorectomy are excluded | Same as in the target trial, except that we restricted to those with continuous health insurance coverage ≥183 days prior to and on the date of trial start (30-day gap in coverage allowed). To mimic a trial where those with fibroids are seeking care, we required individuals to have a recent fibroid claim (recurring or new) within 1 month of trial start |
| **Treatment strategies** | 1. Undergo hysterectomy with bilateral ovarian conservation 2. Undergo myomectomy and no further surgical intervention 3. Do not undergo surgical treatment | Same |
| **Randomized assignment** | Individuals are randomly assigned to one of the three treatment strategies at baseline, and will be aware of the strategy they have been assigned | Participants are assumed to be randomly assigned at baseline within levels of covariates |
| **Start/end of follow up** | Followed from baseline (treatment assignment) until atherosclerotic CVD event, death, loss to follow up, or end of data collection (12/2024), whichever occurs earliest | Same, except mortality data was not available |
| **Outcomes** | 5-year risk of atherosclerotic CVD: Composite of any first-time major atherosclerotic CVD event, defined by the AHA including coronary artery disease (e.g., myocardial infarction), cerebrovascular disease (e.g., ischemic stroke), and peripheral artery disease | Same |
| **Causal contrasts** | Intention-to-treat (**ITT**) effect, i.e., effect of being assigned to undergo myomectomy, hysterectomy, or no surgery at baseline, regardless of whether individuals under surgery after baseline estimated  Per-protocol (**PP**) effect, i.e., effect of being assigned to undergo myomectomy, hysterectomy, or no surgery at baseline, and receiving no further intervention after baseline estimated | Observational analog to the ITT, i.e., the effect of undergoing myomectomy, hysterectomy, or no surgery at baseline, regardless of whether individuals receive surgery after baseline  Observational analog to the PP effect, i.e., the effect of undergoing myomectomy, hysterectomy, or no surgery at baseline, and receiving no further intervention after baseline |
| **Analysis plan** | ITT analysis  PP analysis: We censor data from individuals if/when they deviate from their assigned strategy (i.e., individuals in the no surgery group later undergo a myomectomy or hysterectomy; individuals in the myomectomy group later undergo a hysterectomy). To adjust for potential bias due to censoring at both protocol deviation, we weight the risk estimates by the distribution of the baseline covariates | Same, except estimates are adjusted for baseline covariates |

CVD: cardiovascular disease.

# **Supplemental Figure 1. Schematic Representation of Sequential Target Trial Emulation.**

# **Supplemental Figure 2. Study Population and Cohort Enrollment Process, Optum CDM®** **2000-2019.**

CVD: cardiovascular disease

* Exclusions are not mutually exclusive

# **Supplemental Figure 3. Standardized Mean Differences of Baseline Characteristics Before and After Weighting.**

Models for stabilized inverse probability of treatment weights were estimated using the following covariates, measured at the baseline of each “trial:” age at uterine fibroid diagnosis, age at “trial” start, obesity, hyperlipidemia, Type 2 diabetes mellitus (T2D), hypertension, smoking, obstructive sleep apnea (OSA), Metabolic Dysfunction-associated Fatty Liver Disease (MAFLD), anxiety, depression, parity, infertility, uterine fibroid symptoms (pelvic pain, heavy menstrual bleeding), and prior medical management (oral and non-oral hormonal contraceptives).

# **Supplemental Table 3. 5-Year Regression Results for Secondary Outcomes of Individual Atherosclerotic Cardiovascular Disease Events, Diagnoses, and Risk Factors.**

| **Outcome** | **Treatment** | **Events** | **Unadjusted RD (95% CI) ^a^** | **Adjusted Intention-to-treat RD (95% CI) ^a,b^** | **Adjusted Per-Protocol RD (95% CI) ^a,b,c^** |
| --- | --- | --- | --- | --- | --- |
| Coronary artery disease | Myomectomy | 84 | -0.95 (-1.63, -0.14) | -0.18 (-1.14, 1.63) | -0.17 (-1.05, 1.51) |
|  | Hysterectomy | 285 | 1.15 (0.40, 1.95) | 0.88 (0.03, 1.66) | 0.89 (-0.05, 1.64) |
| Myocardial infarction | Myomectomy | 21 | -0.09 (-0.53, 0.39) | 0.17 (-0.40, 1.33) | 0.17 (-0.44, 1.30) |
|  | Hysterectomy | 74 | 0.60 (0.14, 1.10) | 0.52 (0.02, 1.05) | 0.52 (0.08, 0.96) |
| Cerebrovascular disease | Myomectomy | 66 | -1.23 (-1.89, -0.61) | -0.86 (-1.61, 0.66) | -0.86 (-1.64, 0.42) |
|  | Hysterectomy | 227 | 0.74 (0.05, 1.45) | 0.52 (-0.33, 1.07) | 0.52 (-0.31, 1.18) |
| Ischemic stroke | Myomectomy | 25 | -0.65 (-1.09, -0.20) | -0.42 (-1.00, 0.55) | -0.42 (-1.01, 0.54) |
|  | Hysterectomy | 115 | 0.74 (0.20, 1.38) | 0.62 (-0.06, 1.23) | 0.62 (-0.03, 1.17) |
| Transient ischemic attack | Myomectomy | 33 | -0.18 (-0.57, 0.29) | -0.06 (-0.61, 0.74) | -0.07 (-0.61, 0.79) |
|  | Hysterectomy | 93 | 0.15 (-0.20, 0.54) | 0.04 (-0.37, 0.38) | 0.04 (-0.44, 0.30) |
| Peripheral artery disease | Myomectomy | 11 | -0.05 (-0.33, 0.33) | 0.05 (-0.36, 0.65) | 0.05 (-0.32, 0.74) |
|  | Hysterectomy | 36 | 0.36 (0.04, 0.83) | 0.30 (-0.06, 0.63) | 0.29 (-0.10, 0.60) |
| Obesity | Myomectomy | 1,712 | 2.48 (0.39, 4.43) | 1.44 (-0.87, 3.50) | 1.60 (-0.84, 3.60) |
|  | Hysterectomy | 4,433 | 10.24 (8.72, 11.83) | 9.36 (8.40, 11.73) | 9.50 (8.54, 11.83) |
| Hypertension | Myomectomy | 1,347 | -2.32 (-4.49, -0.31) | -0.33 (-2.83, 3.53) | -0.35 (-2.84, 3.51) |
|  | Hysterectomy | 2,969 | 4.30 (2.51, 5.90) | 3.85 (1.29, 5.62) | 3.89 (1.36, 5.66) |
| Hyperlipidemia | Myomectomy | 1,658 | -4.02 (-6.02, -1.95) | -0.34 (-1.80, 3.80) | -0.43 (-1.92, 3.66) |
|  | Hysterectomy | 3,788 | 5.54 (3.76, 7.15) | 4.45 (1.49, 5.80) | 4.45 (1.50, 5.79) |
| Type 2 Diabetes | Myomectomy | 483 | -2.04 (-3.99, -0.11) | -0.92 (-3.02, 2.41) | -0.92 (-3.01, 2.40) |
|  | Hysterectomy | 1,030 | 0.81 (-0.85, 2.09) | 1.72 (-0.15, 3.48) | 1.70 (-0.16, 3.45) |

RD: risk difference; CI: confidence interval. The reference group is no surgery.

^a^ 95% confidence intervals are estimated using bootstrapping with 500 samples.

^b^ Models included stabilized inverse probability of treatment weights estimated using the following covariates, measured at the baseline of each “trial:” age at uterine fibroid diagnosis, age at “trial” start, obesity, hyperlipidemia, Type 2 diabetes mellitus (T2D), hypertension, smoking, obstructive sleep apnea (OSA), Metabolic Dysfunction-associated Fatty Liver Disease (MAFLD), anxiety, depression, parity, infertility, uterine fibroid symptoms (pelvic pain, heavy menstrual bleeding), and prior medical management (oral and non-oral hormonal contraceptives).

^c^ Per protocol analysis: Models included time-varying stabilized inverse probability weights estimated using baseline and time-varying values of: age at uterine fibroid diagnosis, age at “trial” start, obesity, hyperlipidemia or use of statins, T2D or use of anti-diabetic medications, hypertension or use of antihypertensives, smoking, OSA, MAFLD, anxiety, depression, parity, infertility, uterine fibroid symptoms (pelvic pain, heavy menstrual bleeding), and prior medical management (oral and non-oral hormonal contraceptives).

# **Supplemental Table 4. Subgroup Analyses by Self-Reported Race and Ethnicity.**

| **Race/Ethnicity** | **Treatment** | **Events** | **Unadjusted RD (95% CI)** | **Intention-to-treat RD (95% CI)** |
| --- | --- | --- | --- | --- |
| White | Myomectomy | 60 | -2.99 (-4.68, -1.33) | -2.26 (-4.44, 0.65) |
|  | Hysterectomy | 214 | 0.57 (-0.95, 2.26) | -0.07 (-2.07, 1.24) |
| Black | Myomectomy | 36 | -0.53 (-3.19, 2.46) | 1.29 (-2.86, 8.81) |
|  | Hysterectomy | 117 | 5.10 (2.30, 8.14) | 4.13 (0.43, 7.42) |
| Asian | Myomectomy | 4 | 0.93 (-4.78, 7.55) | -0.52 (-5.95, 4.01) |
|  | Hysterectomy | 7 | 1.07 (-3.99, 8.13) | 0.63 (-4.61, 5.62) |
| Hispanic | Myomectomy | 16 | -1.57 (-5.88, 2.88) | -0.38 (-6.38, 9.55) |
|  | Hysterectomy | 52 | 2.07 (-1.61, 6.67) | 1.52 (-2.98, 5.71) |

RD: risk difference; CI: confidence interval; aRD: adjusted risk difference. The reference group is no surgery.

^a^ 95% confidence intervals are estimated using bootstrapping with 500 samples.

^b^ Models included stabilized inverse probability of treatment weights estimated using the following covariates, measured at the baseline of each “trial:” age at uterine fibroid diagnosis, age at “trial” start, obesity, hyperlipidemia, Type 2 diabetes mellitus (T2D), hypertension, smoking, obstructive sleep apnea (OSA), Metabolic Dysfunction-associated Fatty Liver Disease (MAFLD), anxiety, depression, parity, infertility, uterine fibroid symptoms (pelvic pain, heavy menstrual bleeding), and prior medical management (oral and non-oral hormonal contraceptives).

# **Supplemental Table 5. Subgroup Analyses by Age at Trial Start.**

| **Age at “Trial” Start** | **Treatment** | **Events** | **Unadjusted RD (95% CI)** | **Intention-to-treat RD (95% CI)** |
| --- | --- | --- | --- | --- |
| <40 | Myomectomy | 77 | -0.87 (-2.05, 0.41) | -0.11 (-1.66, 2.73) |
|  | Hysterectomy | 152 | 2.79 (1.23, 4.34) | 1.86 (-0.35, 3.08) |
| ≥40 | Myomectomy | 72 | -1.92 (-3.53, -0.18) | -1.50 (-2.94, 0.74) |
|  | Hysterectomy | 354 | 1.01 (-0.09, 2.16) | 0.91 (-0.31, 2.15) |

RD: risk difference; CI: confidence interval; aRD: adjusted risk difference. The reference group is no surgery.

^a^ 95% confidence intervals are estimated using bootstrapping with 500 samples.

^b^ Models included stabilized inverse probability of treatment weights estimated using the following covariates, measured at the baseline of each “trial:” age at uterine fibroid diagnosis, age at “trial” start, obesity, hyperlipidemia, Type 2 diabetes mellitus (T2D), hypertension, smoking, obstructive sleep apnea (OSA), Metabolic Dysfunction-associated Fatty Liver Disease (MAFLD), anxiety, depression, parity, infertility, uterine fibroid symptoms (pelvic pain, heavy menstrual bleeding), and prior medical management (oral and non-oral hormonal contraceptives).

# **Supplemental Table 6. 5-Year Regression Results for Exploratory Analyses**

| **Analysis** | **Unadjusted Estimate**  **(95% CI)^a^** | **Adjusted Estimate**  **(95% CI)^a,b^** | **Adjusted PP Estimate**  **(95% CI)^a-c^** |
| --- | --- | --- | --- |
| ***Exploratory analysis – Route of surgery*** | | | |
| ⠀⠀RD (Myomectomy – Open/Abdominal vs No Surgery) | -2.24% (-3.62, -0.75) | -1.66% (-3.55, 1.51) | -1.56% (-3.61, 1.87) |
| ⠀⠀RD (Myomectomy – Minimally Invasive vs No Surgery) | -2.20% (-3.41, -0.99) | -1.69% (-2.91, 0.28) | -1.84% (-3.13, 0.42) |
| ⠀⠀RD (Hysterectomy – Open/Abdominal vs No Surgery) | 3.10% (1.56, 4.71) | 2.75% (0.94, 4.06) | 2.93% (1.11, 4.48) |
| ⠀⠀RD (Hysterectomy – Minimally Invasive vs No Surgery) | 0.92% (-0.36, 2.08) | 0.70% (-0.70, 1.91) | 0.86% (-0.39, 2.17) |
| ⠀⠀RR (Myomectomy – Open/Abdominal vs No Surgery) | 0.65 (0.44, 0.88) | 0.74 (0.45, 1.25) | 0.75 (0.43, 1.31) |
| ⠀⠀RR (Myomectomy – Minimally Invasive vs No Surgery) | 0.65 (0.48, 0.84) | 0.73 (0.56, 1.05) | 0.70 (0.52, 1.07) |
| ⠀⠀RR (Hysterectomy – Open/Abdominal vs No Surgery) | 1.49 (1.23, 1.77) | 1.44 (1.14, 1.67) | 1.47 (1.17, 1.72) |
| ⠀⠀RR (Hysterectomy – Minimally Invasive vs No Surgery) | 1.15 (0.95, 1.35) | 1.11 (0.89, 1.32) | 1.14 (0.94, 1.37) |
| ***Exploratory analysis - Oophorectomy*** | | | |
| ⠀⠀RD (Myomectomy vs No Surgery) | -2.60% (-3.51, -0.92) | -1.27% (-2.53, 1.76) | -1.83% (-2.92, 1.42) |
| ⠀⠀RD (Hysterectomy vs No Surgery) | 1.60% (0.30, 2.89) | 1.12% (-0.43, 2.09) | 0.71% (-0.86, 1.96) |
| ⠀⠀RD (Hysterectomy with unilateral oophorectomy vs No Surgery) | 2.98% (0.32, 5.86) | 2.06% (-0.87, 4.60) | 1.64% (-1.09, 4.74) |
| ⠀⠀RR (Myomectomy vs No Surgery) | 0.67 (0.53, 0.86) | 0.84 (0.65, 1.28) | 0.78% (0.60, 1.21) |
| ⠀⠀RR (Hysterectomy vs No Surgery) | 1.20 (1.04, 1.45) | 1.14 (0.94, 1.35) | 1.09 (0.89, 1.33) |
| ⠀⠀RR (Hysterectomy with unilateral oophorectomy vs No Surgery) | 1.37 (1.04, 1.89) | 1.26 (0.88, 1.77) | 1.20 (0.85, 1.69) |

RD: risk difference; RR: risk ratio; CI: confidence interval

^a^ 95% confidence intervals are estimated using bootstrapping with 500 samples.

^b^ Models included stabilized inverse probability of treatment weights estimated using the following covariates, measured at the baseline of each “trial:” age at uterine fibroid diagnosis, age at “trial” start, obesity, hyperlipidemia, Type 2 diabetes mellitus (T2D), hypertension, smoking, obstructive sleep apnea (OSA), Metabolic Dysfunction-associated Fatty Liver Disease (MAFLD), anxiety, depression, parity, infertility, uterine fibroid symptoms (pelvic pain, heavy menstrual bleeding), and prior medical management (oral and non-oral hormonal contraceptives).

^c^ Per protocol analysis: Models included time-varying stabilized inverse probability weights estimated using baseline and time-varying values of: age at uterine fibroid diagnosis, age at “trial” start, obesity, hyperlipidemia or use of statins, T2D or use of anti-diabetic medications, hypertension or use of antihypertensives, smoking, OSA, MAFLD, anxiety, depression, parity, infertility, uterine fibroid symptoms (pelvic pain, heavy menstrual bleeding), and prior medical management (oral and non-oral hormonal contraceptives).

# **Supplemental Table 7. Sensitivity Analyses for Atherosclerotic Cardiovascular Disease Risk.**

| **5-year Risk Differences, % (95% CI) ^a,b^** | **Primary** | **Exclude individuals with infertility codes** | **Require ≥ 12 months baseline data** | **Censoring weights for disenrollment ^c^** |
| --- | --- | --- | --- | --- |
| Myomectomy | -0.93%  (-2.04, 1.30) | -0.92  (-2.19, 1.41) | -1.12  (-2.33, 0.9) | -0.81  (-2.54, 1.89) |
| Hysterectomy | 1.24%  (-0.01, 2.03) | 1.16  (-0.05, 1.89) | 1.12  (-0.07, 1.9) | 2.02  (0.29, 3.04) |
| No surgery | (ref) | (ref) | (ref) | (ref) |

The reference group is no surgery.

^a^ 95% confidence intervals are estimated using bootstrapping with 500 samples.

^b^ Models included stabilized inverse probability of treatment weights estimated using the following covariates, measured at the baseline of each “trial:” age at uterine fibroid diagnosis, age at “trial” start, obesity, hyperlipidemia, Type 2 diabetes mellitus (T2D), hypertension, smoking, obstructive sleep apnea (OSA), Metabolic Dysfunction-associated Fatty Liver Disease (MAFLD), anxiety, depression, parity, infertility, uterine fibroid symptoms (pelvic pain, heavy menstrual bleeding), and prior medical management (oral and non-oral hormonal contraceptives).

^c^ Loss to follow-up analysis: Models included time-varying stabilized inverse probability weights estimated using baseline and time-varying values of: age at uterine fibroid diagnosis, age at “trial” start, obesity, hyperlipidemia or use of statins, T2D or use of anti-diabetic medications, hypertension or use of antihypertensives, smoking, OSA, MAFLD, parity, infertility or use of infertility medications, uterine fibroid symptoms, prior medical management.

# **Supplemental Table 8. 5-Year Regression Results for Sensitivity Analyses of Surgical Route**

| **Analysis** | **Unadjusted Estimate**  **(95% CI)^a^** | **Adjusted Estimate**  **(95% CI)^a,b^** | **Adjusted PP Estimate**  **(95% CI)^a-c^** |
| --- | --- | --- | --- |
| ⠀⠀RD (Myomectomy – Open/Abdominal vs No Surgery) | -2.24% (-3.54, -0.61) | -1.64% (-3.52, 1.80) | -1.53% (-3.55, 2.14) |
| ⠀⠀RD (Myomectomy – Minimally Invasive vs No Surgery) | -2.14% (-3.98, -0.28) | -1.49% (-4.05, 2.64) | -1.49% (-4.23, 3.42) |
| RD (Myomectomy – Hysteroscopic vs No Surgery) | -2.22% (-3.92, -0.99) | -1.99% (-3.50, -0.28) | -1.88% (-3.40, -0.04) |
| ⠀⠀RD (Hysterectomy – Open/Abdominal vs No Surgery) | 3.10% (1.50, 4.49) | 2.72% (0.94, 4.06) | 2.90% (1.08, 4.41) |
| ⠀⠀RD (Hysterectomy – Minimally Invasive vs No Surgery) | 0.92% (-0.26, 2.17) | 0.70% (-0.57, 1.88) | 0.86% (-0.54, 2.15) |
| ⠀⠀RR (Myomectomy – Open/Abdominal vs No Surgery) | 0.65 (0.44, 0.90) | 0.74 (0.45, 1.30) | 0.76 (0.44, 1.36) |
| ⠀⠀RR (Myomectomy – Minimally Invasive vs No Surgery) | 0.66 (0.40, 0.96) | 0.76 (0.37, 1.40) | 0.76 (0.32, 1.55) |
| RR (Myomectomy – Hysteroscopic vs No Surgery) | 0.65 (0.41, 0.84) | 0.69 (0.47, 0.95) | 0.70 (0.47, 0.99) |
| ⠀⠀RR (Hysterectomy – Open/Abdominal vs No Surgery) | 1.49 (1.24, 1.71) | 1.43 (1.15, 1.64) | 1.46 (1.17, 1.74) |
| ⠀⠀RR (Hysterectomy – Minimally Invasive vs No Surgery) | 1.15 (0.96, 1.38) | 1.11 (0.91, 1.30) | 1.14 (0.92, 1.37) |

RD: risk difference; RR: risk ratio; CI: confidence interval

^a^ 95% confidence intervals are estimated using bootstrapping with 500 samples.

^b^ Models included stabilized inverse probability of treatment weights estimated using the following covariates, measured at the baseline of each “trial:” age at uterine fibroid diagnosis, age at “trial” start, obesity, hyperlipidemia, Type 2 diabetes mellitus (T2D), hypertension, smoking, obstructive sleep apnea (OSA), Metabolic Dysfunction-associated Fatty Liver Disease (MAFLD), anxiety, depression, parity, infertility, uterine fibroid symptoms (pelvic pain, heavy menstrual bleeding), and prior medical management (oral and non-oral hormonal contraceptives).

^c^ Per protocol analysis: Models included time-varying stabilized inverse probability weights estimated using baseline and time-varying values of: age at uterine fibroid diagnosis, age at “trial” start, obesity, hyperlipidemia or use of statins, T2D or use of anti-diabetic medications, hypertension or use of antihypertensives, smoking, OSA, MAFLD, anxiety, depression, parity, infertility, uterine fibroid symptoms (pelvic pain, heavy menstrual bleeding), and prior medical management (oral and non-oral hormonal contraceptives).
