# Supplementary material for: Emulating a target trial of surgical removal of uterine fibroids on atherosclerotic cardiovascular disease
Source: Fertil Steril. Author manuscript; Available in PMC 2026 Oct 2. (PMC13631833; doi:10.1016/j.fertnstert.2026.05.151)
Supplement: Supplementary file 2 [file NIHMS2212450-supplement-2.docx]

Table of Contents

[Supplemental Appendix 1. Additional details on the data source.	2](#_Toc210039110)

[Supplemental Appendix 2. Methodological details for weighting methods.	3](#_Toc210039111)

[A.	Inverse Probability of Treatment Weights (IPTW)	3](#_Toc210039112)

[B.	Weighting For Selection Bias due to Artificial Censoring: Per Protocol Effect	4](#_Toc210039113)

[C.	Cumulative Weights Used in Models	4](#_Toc210039114)

[D.	Sensitivity Analysis Weighting for Selection Bias: Disenrollment in Optum	4](#_Toc210039115)

# **Supplemental Appendix 1. Additional Details on the Data Source.**

The analyses were conducted in Optum’s de-identified Clinformatics® Data Mart Database (Optum CDM®). Optum CDM® is derived from a database of administrative health claims for members of large commercial and Medicare Advantage health plans. Optum CDM® administrative claims are submitted for payment by providers and pharmacies are verified, adjudicated and de-identified prior to inclusion. These data, including patient-level enrollment information, are derived from claims submitted for all medical and pharmacy health care services with information related to health care costs and resource utilization.

Data is included for only those covered lives with both medical and prescription drug coverage, to enable users to evaluate the claims related to the complete health care experience. The population is geographically diverse, spanning all 50 states. Enrollees have unique member identifiers, facilitating longitudinal study of claims over time. The internal database is updated monthly.

Given the age range of our analysis (18–50-year-old at baseline and followed for 5 years), the cohort for this study was privately insured individuals. Optum CDM® are considered highly generalizable, given that approximately 65% of the US population is covered by a private insurer.

# **Supplemental Appendix 2. Methodological Details for Weighting Methods.**

## A. Inverse Probability of Treatment Weights (IPTW)

To account for measured confounding by baseline differences between the three treatment groups, we estimated stabilized inverse probability of treatment weights (IPTWs). We fit logistic regression models to extract predicted probabilities for the numerator and denominator of the weights. The numerator modeled the marginal probability of receiving each treatment strategy (myomectomy, hysterectomy, or no surgery). The denominator modeled the probability of receiving each treatment strategy conditional on a vector of baseline covariates, outlined below:

| **Group** | **Covariate** | **Variable Type** | **Source** |
| --- | --- | --- | --- |
| Demographic | Age at uterine fibroid diagnosis | Continuous, Modeled using natural splines | Calculated from year of first uterine fibroid claim and individual year of birth |
| Demographic | Age at “trial” start | Continuous, Modeled using natural splines | Calculated from “trial” start and individual year of birth |
| Medical comorbidities | Obesity | Binary: Yes, No | ICD-9 and 10; CPT/HCPCS |
| Medical comorbidities | Hyperlipidemia or use of statins | Binary: Yes, No | ICD-9 and 10; CPT/HCPCS; NDC codes |
| Medical comorbidities | Type 2 diabetes or use of diabetes medications | Binary: Yes, No | ICD-9 and 10; CPT/HCPCS; NDC codes |
| Medical comorbidities | Hypertension or use of antihypertensives | Binary: Yes, No | ICD-9 and 10; CPT/HCPCS; NDC codes |
| Medical comorbidities | Smoking | Binary: Yes, No | ICD-9 and 10; CPT/HCPCS |
| Medical comorbidities | Anxiety or use of anxiolytics | Binary: Yes, No | ICD-9 and 10; CPT/HCPCS; NDC codes |
| Medical comorbidities | Depression or use of antidepressants | Binary: Yes, No | ICD-9 and 10; CPT/HCPCS; NDC codes |
| Reproductive history | Parity | Binary: Yes, No | ICD-9 and 10; CPT/HCPCS |
| Reproductive history | Infertility or use of clomiphene or gonadotropins | Binary: Yes, No | ICD-9 and 10; CPT/HCPCS |
| Reproductive history | Endometriosis | Binary: Yes, No | ICD-9 and 10; CPT/HCPCS |
| Uterine fibroid pathology and treatment | Symptoms (pelvic pain or heavy menstrual bleeding) | Binary: Yes, No | ICD-9 and 10; CPT/HCPCS |
| Uterine fibroid pathology and treatment | Hormonal contraceptive use | Binary: Yes, No | NDC codes |

Individuals in the myomectomy group received weights, ${sw}_{A}=\frac{\Pr\left[ A=1 \right]}{Pr[A=1|L]}$, where $A$ is a treatment indicator ($A=1$ for myomectomy, $A=2$ for hysterectomy; $A=0$ for no surgery) and $L$ is the vector of covariates. Individuals in the hysterectomy group received weights, ${sw}_{A}=\frac{\Pr\left[ A=2 \right]}{Pr[A=2|L]}$. Individuals in the no surgery group received weights, ${sw}_{A}=\frac{\Pr\left[ A=0 \right]}{Pr[A=0|L]}$. These weights accounted for measured confounding by treatment strategy at baseline only.

## B. Weighting For Selection Bias due to Artificial Censoring: Per Protocol Effect

In the per-protocol analysis, individuals in the myomectomy group are censored if and when they receive a hysterectomy. Similarly, individuals in the no surgery group are censored if and when they receive a myomectomy or hysterectomy. To address potential selection bias due to artificial censoring, we weighted each individual at each monthby the inverse probability of receiving their own time-varying treatment history.

For those in the myomectomy group where $A=1$, ${sw}_{t}^{V}=\prod_{k=0}^{t} \frac{Pr(A_{k}=1 |\bar{A}_{k-1}, L_{0}, D_{k}=0)}{Pr(A_{k}=1 |\bar{A}_{k-1},\bar{L}_{k}, D_{k}=0)}$. For those in the hysterectomy group where $A=2$, ${sw}_{t}^{V}=\prod_{k=0}^{t} \frac{Pr(A_{k}=2|\bar{A}_{k-1}, L_{0}, D_{k}=0)}{Pr(A_{k}=2|\bar{A}_{k-1},\bar{L}_{k}, D_{k}=0)}$. For those in the no surgery group where $A=0$, ${sw}_{t}^{V}=\prod_{k=0}^{t} \frac{Pr(A_{k}=0|\bar{A}_{k-1}, L_{0}, D_{k}=0)}{Pr(A_{k}=0|\bar{A}_{k-1},\bar{L}_{k},D_{k}=0)}$.

For an individual who is uncensored through month *t* (i.e., they did not switch treatments), note that the contribution to the denominator of the weight is equal to the probability that the individual remains uncensored through month *t* conditional on not developing the outcome by month *t*, covariate history, and treatment history. There are two variables in reference to time: *t* refers to the weight (cumulative product) at each time, while *k* is the contribution to that weight at each time. $\bar{A}_{k-1}$ denotes treatment history. $\bar{L}_{k}$ denotes baseline and current values of covariates.

An individual’s weight at each time is the cumulative product of the conditional probability of remaining uncensored. The weights are estimated by fitting a pooled logistic model including a time-specific intercept, the baseline covariates, and the most recent measurement of several time-varying covariates (see table above).

## C. Cumulative Weights Used in Models

For the ITT effect, an individual’s IPW at each month they are observed in the dataset is ${sw}_{A}$, which is the IPTW. For the per-protocol effect, an individual’s IPW at each month they are observed in the dataset is ${sw}_{A}{\times sw}_{t}^{V},$ which is the product of the IPTW and the IPW for artificial censoring.

## D. Sensitivity Analysis Weighting for Selection Bias: Disenrollment in Optum CDM®

In administrative claims data, it is common for individuals to enroll and disenroll in a health plan. To account for potential selection bias due to individuals disenrolling in Optum CDM®, we used stabilized inverse probability of censoring weights (IPCW) in sensitivity analyses.

First, we fit logistic regression models to extract predicted probabilities of remaining enrolled in Optum CDM® at each month of follow-up for the numerator and denominator of the weights. The numerator modeled the marginal probability of remaining enrolled in Optum CDM® at month $t$, conditional on baseline exposure status, $A$. The denominator modeled the probability of remaining enrolled in Optum at month $t$, conditional on baseline exposure status, $A$, and a vector of covariates updated at month $t$outlined above. The overbar represents an individual’s covariate, treatment, or censoring history.

Those who remained enrolled in Optum CDM® at month $t$ receive weights, ${sw}_{t}^{C}=\prod_{k=0}^{t} \frac{Pr(C_{k+1}=0|\bar{C}_{k}=0, \bar{A}_{k}, L_{0}, D_{k}=0)}{Pr(C_{k+1}=0|\bar{C}_{k}=0, \bar{A}_{k},\bar{L}_{k}, D_{k}=0)}$, and those who disenroll in Optum CDM® at month $t$ receive a weight, ${sw}_{t}^{C}=0$. In this sensitivity analysis, an individual’s IPW at each month they are observed in the dataset is ${sw}_{A}{\times sw}_{t}^{V}\times{sw}_{t}^{C},$ which is the product of the IPTW, IPW for artificial censoring, and IPCW.
